# Performance of Impedance Control-Based Strategies in Power-Assisted Wheelchairs: A Predictive Simulation Study

**DOI:** 10.3389/fnbot.2022.805835

**Published:** 2022-03-04

**Authors:** Vinicius Ishimoto Cuerva, Marko Ackermann, Fabrizio Leonardi

**Affiliations:** Department of Mechanical Engineering, Centro Universitário FEI, São Bernardo do Campo, Brazil

**Keywords:** power-assisted wheelchairs, impedance control, optimal control, predictive simulations, assistive technology

## Abstract

Manual wheelchair propulsion is known to be inefficient and causes upper extremity pain, fatigue, and injury. Power-assisted wheelchairs can mitigate these effects through motors that reduce users' effort and load during propulsion. Among the different control strategies proposed to govern the user-wheelchair interaction, impedance control-based ones appear to be the most natural and effective. It can change the apparent dynamical properties of the wheelchair, particularly mass and friction, and automatically compensate for external disturbances such as terrain conditions. This study investigates the advantages and disadvantages of this control strategy employing predictive simulations of locomotion with power-assisted wheelchairs in different scenarios. The simulations are generated using a biomechanically realistic model of the upper extremities and their interaction with the power-assisted wheelchair by solving an optimal control problem. Investigated scenarios include steady-state locomotion vs. a transient maneuver starting from rest, movement on a ramp vs. a level surface, and different choices of reference model parameters. The results reveal that the investigated impedance control-based strategy can effectively reproduce the reference model and reduce the user's effort, with a more significant effect of inertia in the transient maneuver and of friction in steady-state locomotion. However, the simulations also show that imposing a first-order, linear reference model with constant parameters can produce disadvantageous locomotion patterns, particularly in the recovery phase, leading to unnecessary energy dissipation and consequent increase in energy consumption from the batteries. These observations indicate there is room for improvement, for instance, by exploring energy regeneration in the recovery phase or by switching reference model nature or parameters along the cycle. To the best of our knowledge, this is the first investigation of impedance control-based strategies for power-assisted wheelchairs using predictive simulations and a realistic, nonlinear model of the user-wheelchair system.

## 1. Introduction

Wheelchair locomotion is common among people with physical disabilities and can help them have a more independent living. However, locomotion with manual, pushrim-propelled wheelchairs is known to have low efficiency and to impose significant and repetitive loads on the upper extremity, often leading to muscle fatigue, pain and injuries (van der Woude et al., 2001).

In order to mitigate these effects, wheelchairs with partial motor assistance are drawing increasing interest, and studies compare the performance of commercially available models in different circumstances (Karmarkar et al., 2008). This type of wheelchair is often referred to as PAPAW (Pushrim-Activated Power Assisted Wheelchairs). It has electric motors that assist the person in manually propelling the wheelchair, thus enabling the user to exercise while avoiding excessive muscle effort and upper extremity loads and, consequently, reducing the risk of muscle fatigue and joint injuries (Kloosterman et al., 2012). This type of assistance is important in conditions where large torques are required, such as on ramps or rough terrain (Kloosterman et al., 2012). It can also be helpful on descents where the assistance torque can act as a brake to ensure safer locomotion (Seki et al., 2009).

There are different types of assistance strategies for a PAPAW. The simplest are those based on feedforward control laws where the motor torque is directly related to the torque applied by the user on the pushrim. The most elementary version of this type of assistance applies constant torque whenever the pushrim is actuated by the user.

A frequently used feedforward strategy is the generation of an assistance torque proportional to the torque applied by the user (Guillon et al., 2015), but this torque amplification strategy may compromise handling of the wheelchair due to possible differences in the magnitudes of forces applied by the person's right and left arms on the pushrim (Heo et al., 2018). In order to provide an effective shared control system, Cooper et al. (2002) propose a proportional feedforward control during the propulsion phase, a linear decay of assistance over time in the recovery phase, and a regenerative braking in case the maximum speed threshold is achieved.

The literature reports a series of other control strategies for PAPAWs that range from reducing the effects of the environment on the dynamics of wheelchairs to the assistance in particular conditions and applications. Lee et al. (2016), for instance, attempt to correct for the effects of gravity on ramps by introducing inclination sensors and requiring additional information such as user's mass. Oonishi et al. (2010), in turn, proposes the combined use of an electromyography-based estimator of user intention and a disturbance torque estimator to define the assistance torque. Assistance strategies are also proposed for special maneuvers such as steps climbing (Seki et al., 2006) and wheelie (Santos et al., 2016).

Among the various control strategies proposed for power-assisted wheelchairs, a promising one is the impedance control as it provides a natural interaction with the user by manipulation of the apparent system properties, such as apparent mass and friction. In fact, impedance control is a well-established technique to control the relationship between the movement kinematics and the force between robots and humans (Hogan, 1985) and is widely used in situations where the environment influences the controlled dynamic system, such as in exoskeletons (Li et al., 2017). Its application to power-assisted wheelchairs is investigated in different studies (Oh and Hori, 2014; Shieh et al., 2015; Lee et al., 2016).

The implementation of the impedance control strategy often requires the adoption of a reference model for the wheelchair system, which is almost invariably assumed as a linear first-order model composed of a lumped mass and a viscous damping. It is, indeed, a common practice to investigate and design assistance strategies for power-assisted wheelchairs on the basis of such simple mass-damper models (Chénier et al., 2014; Oh and Hori, 2014; Shieh et al., 2015; Lee et al., 2016; Heo et al., 2018). Such first-order models, however, neglect the dynamics associated with the cyclic motion of the arms and the biomechanics of the upper extremity, which may lead to substantial inaccuracies in representing the wheelchair-user system dynamics (Chénier et al., 2014).

Assessing the performance of impedance control-based strategies in different locomotion conditions, investigating the potentially deleterious effects of using a simple mass-damper model as a reference model, is an important step toward its effective implementation in real power-assisted wheelchairs. Considering this, the main objective of this study is to evaluate, through predictive simulations of wheelchair locomotion at different typical conditions, the performance of assistance based on impedance control and the effects of considering in the control law a first-order dynamics (mass/damper) as the reference model. The proposed simulation framework is based on a musculoskeletal model of the upper extremities and its interaction with the power-assisted wheelchair, and the solution of an optimal control problem. The effects of alterations in the reference model parameters are predicted for steady-state and transient locomotion on a level surface and on a ramp.

## 2. Materials and Methods

The approach adopted here is similar to that in Ackermann et al. (2014). A biomechanical model of the upper extremity and its interaction with the wheelchair is used to represent the two phases of the wheelchair locomotion cycle. An impedance control strategy is implemented whose objective is that the system dynamics seen by the user matches a reference, linear, lumped-mass model subject to viscous damping. Different mass and damping combinations are tested to investigate the effects on system performance in terms of muscle and motor effort. Simulations are generated by solving an optimal control problem to predict the performance in four locomotion conditions representing different activities of daily living, steady-state and transient locomotion on level surfaces and ramps.

### 2.1. Wheelchair-User Model

**Figure 1:**
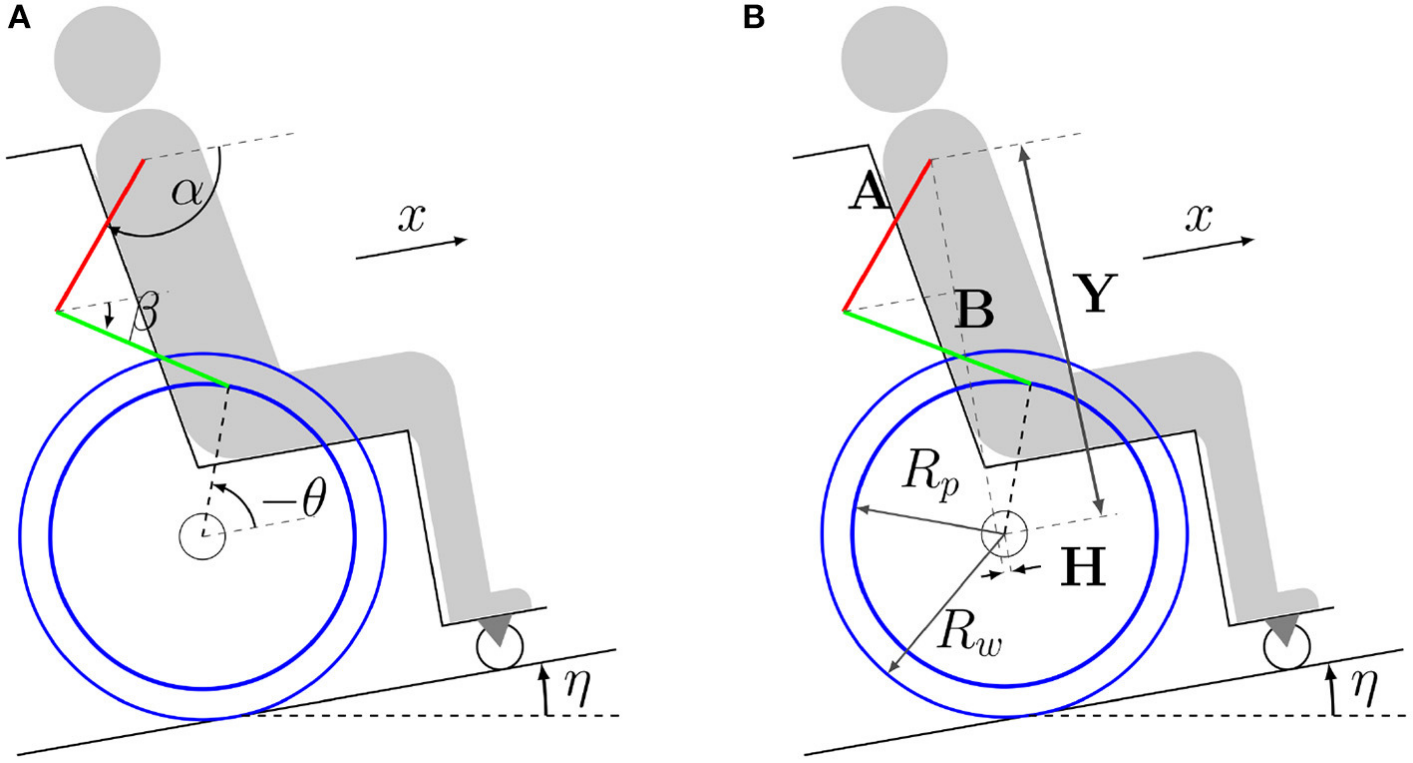
Model of the wheelchair-user system composed of four rigid bodies representing arms (red), forearms (green), rear wheels (blue) and the remaining body and wheelchair elements (gray/black). Coordinates are represented in figure **(A)**, where α is the upper arm angle with respect to the direction of motion, β is the forearm angle with respect to the direction of motion, θ is the rear wheel angular displacement, *x* is the forward displacement of the shoulder/wheelchair and η is the ramp inclination angle. Lengths are represented in figure **(B)**, where *Y* is the relative vertical and *H* is the relative horizontal distance between the center of the rear wheels and the shoulder, *A* is the upper arm length, *B* is the forearm length, *R*_*p*_ is the pushrim radius, and *R*_*w*_ is the rear wheel radius.

A moving four-bar mechanism is adopted to model the wheelchair-user system (Ackermann et al., 2014). Bilateral symmetry is assumed, which is usual in daily wheelchair locomotion, (Goosey-Tolfrey and Kirk, 2003; Soltau et al., 2015). The user model represents an average individual, whose total mass (70.0*kg*) and height (1.70*m*) are consistent with Brazilian male averages and with ranges reported in Gil-Agudo et al. (2010). The model is formed by four rigid bodies representing arms, forearms, rear wheels, and a fourth rigid body encompassing the remaining segments of the body and elements of the wheelchair, as illustrated in Figure 1.

The shoulder and elbow joints and the wheel axle are considered ideal hinge joints. In the recovery phase, in which the hand is not in contact with the pushrim and undergo repositioning in preparation for the next propulsion phase, the model has three degrees of freedom, and the generalized coordinate vector is


(1) $$q=\left[\begin{array}{c} \alpha \\ \beta \\ \theta \end{array}\right]\;,$$

where α and β are the angles between forearms and arms concerning the direction of motion, respectively, and θ is the angular displacement of the wheelchair's rear wheels, as shown in Figure 1. In the propulsion phase, the contact between the hands and the pushrims is represented by a hinge joint that transforms the mechanical system into a moving four-bar mechanism with a single degree of freedom.

Applying the D'Alembert's principle in a code implemented in MATLAB, we derived the equations of motion in their minimal form for the recovery phase as


(2) $$\begin{array}{l} M\left(q\right)\ddot{q}+k\left(q,\overset{\cdot }{q}\right)=k_{e}\left(q\right), \end{array}$$

where *M* is the mass matrix, *k* is the vector of generalized Coriolis and centrifugal forces and *k*_*e*_ is the generalized force vector.

Length and inertia parameters are determined for a person 1.70 m in height with a total body mass of 70 kg, based on anthropometric data in Winter (2009), see Table 1. As the simulations do not involve turning, the only resistive force considered was the rolling resistance (Sauret et al., 2012), adopted as 20 N, corresponding to an approximate value reported in van der Woude et al. (2001) for locomotion on a vinyl pavement.

**Table 1:** Adopted model parameters.

**Dimensions**	
User's height	1.70 m
Upper arm length (*A*)	0.3162 m
Upper arm center of mass location	0.1379 m
Forearm length (*B*)	0.3400 m
Forearm center of mass location	0.1693 m
Pushrim radius (*R*_*p*_)	0.2570 m
Rear wheel radius (*R*_*w*_)	0.2988 m
Rear-wheel/shoulder distance (*H*)	0.05 m
Rear-wheel/shoulder distance (*Y*)	0.75 m
**Inertia properties**	
User's mass	70.0 kg
Upper arms mass	3.9200 kg
Upper arms moment of inertia	0.0406 kg m^2^
Forearms mass	3.0800 kg
Forearms moment of inertia	0.0416 kg m^2^
Rear wheels moment of inertia	0.1274 kg m^2^
Combined mass (Wheelchair+user)	72.5200 kg

In the propulsion phase, in which a hinge joint is formed between the hands and the pushrims, the generalized coordinates α, β and θ are linked through the holonomic constraints


(3) $$c(\alpha ,\beta ,\theta )=\left[\begin{array}{c} A\text{ cos }\alpha +B\text{ cos }\beta -R_{p}\text{ cos }\theta -H\\ A\text{ sin }\alpha +B\text{ sin }\beta -R_{p}\text{ sin }\theta -Y \end{array}\right]=0,$$

where *H* and *Y* are the horizontal and vertical distances between the shoulder and the center of the wheelchair's rear wheels, respectively, *A* is the upper arm length, *B* is the forearm length and *R*_*p*_ is the pushrim radius, whose values are adopted as indicated in Table 1.

Considering the constraints in Equation (3), the equations of motion in the minimal form for the propulsion phase are obtained from Equation (2) as


(4) $$\begin{array}{l} J^{T}MJ\ddot{\theta }+J^{T}M\frac{dJ}{dt}\overset{\cdot }{\theta }+J^{T}k=J^{T}k_{e}, \end{array}$$

where *J* is the Jacobian defined as


(5) $$\begin{array}{ll} J\left(\alpha ,\beta ,\theta \right) & =\frac{\partial q}{\partial \theta }. \end{array}$$

Using ideal joint torque generators can lead to unrealistic joint torque patterns, such as torque discontinuities between phases, unrealistic torque magnitudes, and torque peaks when the elbow is close to full extension, due to the large mechanical gain in this configuration. For these reasons, it is important to take the physiological, intrinsic musculoskeletal properties into account. Muscle force-length and force-velocity relationships and passive joint torques due to ligaments and connective tissue were adopted from Brown (2018), that reports data obtained for an elite wheelchair basketball athlete. According to this approach, upper extremity joint torques in both, extension and flexion, are functions of elbow and shoulder angles and angular velocities as


(6) $$\begin{array}{l} \tau_{i}=f_{i}\left(a_{i},\alpha ,\overset{\cdot }{\alpha },\beta ,\overset{\cdot }{\beta }\right), \end{array}$$

where *a*_*i*_ ∈ [0, 1] represents equivalent, global muscle activations for shoulder extensors (*i* = *se*), shoulder flexors (*i* = *sf*), elbow extensors (*i* = *ee*) and elbow flexors (*i* = *ef*) that modulate the corresponding active joint torques τ_*i*_.

The muscle activation dynamic is modeled, as in He et al. (1991), by a linear, first-order dynamic as


(7) $$\begin{array}{l} \frac{da_{i}}{dt}=\left(u_{i}-a_{i}\right)\left(\frac{u_{i}}{T_{a}}+\frac{1-u_{i}}{T_{d}}\right), \end{array}$$

where *u*_*i*_ ∈ [0, 1] is the neural excitation corresponding to the muscle activation *a*_*i*_, *T*_*a*_ is the activation time constant assumed as 20 ms and *T*_*d*_ is the deactivation time constant assumed as 40 ms. The adopted activation and deactivation time constants are consistent with values reported in the literature, as in Brown (2018).

### 2.2. Impedance Control

The assistance strategy considered is continuous and based on the impedance control, which aims at imposing the dynamic behavior between the force applied by the user and the wheelchair velocity, i.e., the dynamics seen by the user. The chosen reference dynamics are commonly used in the literature, corresponding to a lumped mass and a resistive damping force. This dynamics is the impedance control reference model, as


(8) $$\begin{array}{l} M_{i}\frac{dv_{r}}{dt}+C_{i}v_{r}=\frac{\tau_{p}}{R_{w}}, \end{array}$$

where *M*_*i*_ is the apparent mass, *C*_*i*_ is the apparent damping coefficient, τ_*p*_ is the torque applied by the user, *R*_*w*_ is the wheel radius and *v*_*r*_ is the reference speed for the control loop, with *v*_*r*_ = *R*_*w*_ω_*r*_.

The nominal parameters of this reference model, *M*_*i*_ and *C*_*i*_, are obtained based on a linear approximation of the four-bar model in Figure 1. The parameters that best fit the response of the nonlinear model for a startup simulation on level ground in open loop are identified using the *tfest* MATLAB transfer function estimator.

**Figure 2:**
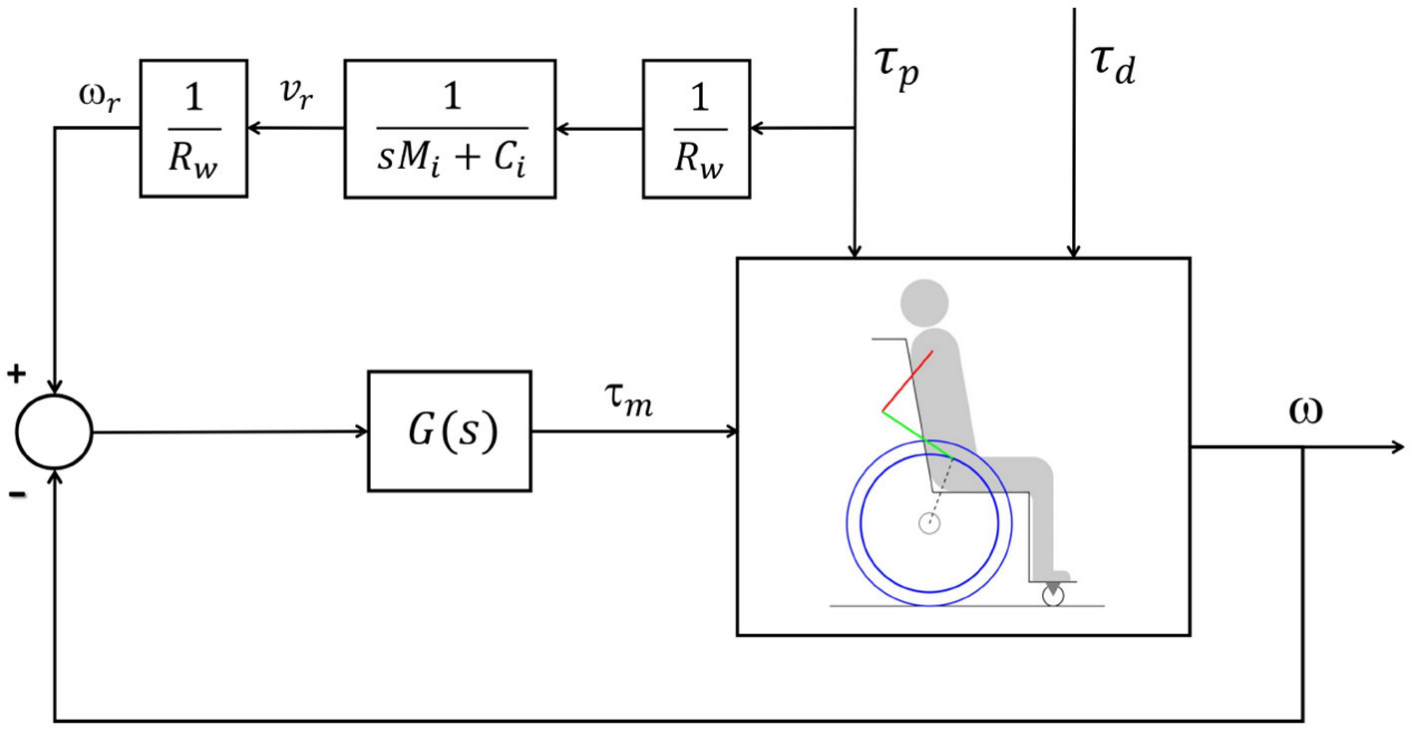
Block diagram representing the impedance control loop, where the signal τ_*p*_ is the user torque, τ_*d*_ is the disturbance torque, τ_*m*_ is the motor torque, *v*_*r*_ is the reference rear wheel velocity generated by the reference model, ω_*r*_ is the reference rear wheel angular velocity, and ω is the angular velocity of the wheelchair rear wheels. The transfer function *G*(*s*) represents the velocity controller. The parameter *R*_*w*_ is the wheel radius, *M*_*i*_ is the desired apparent mass and *C*_*i*_ is the desired apparent friction coefficient.

In order to indirectly impose the apparent impedance, feedback control is employed to control the speed based on the desired speed generated by the reference impedance. The control strategy adopted is illustrated by the block diagram in Figure 2, where G(s) is a PI controller that seeks to impose the reference angular velocity ω_*r*_ to the output angular velocity ω. For this purpose, the controller is designed using the pole and zero cancellation technique, resulting in a closed-loop system that has similar behavior to the first-order system in Equation (8). The time constant of this closed loop was adjusted to 0.02 s so that its dynamics would be negligible compared to the other time constants of the wheelchair-user system dynamics.

The reference angular velocity ω_*r*_ is produced based on the torque τ_*p*_ applied by the user to the pushrim and through the first-order reference model that describes the desired mechanical impedance in the sense that, if the speed control error is small, so the relationship between ω and τ_*p*_ is apparently imposed in a similar way to that of the reference impedance.

### 2.3. Predictive Simulation Approach

The predictive simulations are generated by solving an optimal control problem, a formulation commonly used in studies on human movement in activities such as walking (Ackermann and van den Bogert, 2010; Sreenivasa et al., 2017), jumping (Porsa et al., 2015), and also wheelchair locomotion (Brown and McPhee, 2020).

The predictive simulations for all conditions and scenarios consisted of searching optimal motion duration *t*_*f*_ and time series of the controls (equivalent neural excitations) and states (equivalent muscle activations and generalized coordinates and their time derivatives) that minimize a cost function quantifying physiological muscle effort as


(9) $$W_{p}=\int_{0}^{t_{f}}\left(u_{ee}^{2}+u_{ef}^{2}+u_{se}^{2}+u_{sf}^{2}\right)\;dt,$$

where the subindices *se* refers to the shoulder extensors, *sf* to the shoulder flexors, *ee* to the elbow extensors, and *ef* to the elbow flexors.

The performance of the control strategy in the different conditions and reference model parameter combinations was assessed in terms of user and motor effort. User effort was quantified by *W*_*p*_ in Equation (9), which is the cost function minimized to generate the predictive simulations of unassisted and assisted locomotion. The motor effort, in turn, is quantified by


(10) $$W_{m}=\int_{0}^{t_{f}}\left(\tau_{m}^{2}\right)\;dt.$$

The constraints of the optimal control problem include the dynamics in Equation (2) for the recovery phases, in Equation (4) for the propulsion phases, and in Equation (7) for the activation dynamics. In the assisted locomotion simulations, in which the control loop in Figure 2 is active, the corresponding closed-loop dynamics is included as well. Other constraints are: continuity of all states between adjacent propulsion and recovery phases, the imposition of an average speed of 0.9 m/s, and physiological upper and lower bounds on neural excitations (*u*_*i*_ ∈ [0, 1]), muscle activations (*a*_*i*_ ∈ [0, 1]) and generalized coordinates and velocities.

In the *steady state* simulations, periodicity constraints are added on initial and final states of one complete cycle to ensure a periodic motion at a prescribed average speed, with one propulsion phase and one recovery phase. There are no periodicity constraints in the *startup* simulations, the wheelchair starts from rest, with ω(t=0)=0, and a prescribed total displacement of 1.6 m is added. The *startup* simulation is composed of five phases in the following sequence: propulsion-recovery-propulsion-recovery-propulsion.

All simulations are performed using the direct collocation method and implemented in MATLAB. The time discretization was performed using the pseudospectral Radau method (Garg et al., 2009) where the derivative of the states are obtained by deriving a Lagrange polynomial. The resulting nonlinear optimization problem (NLP) is then solved using the IPOPT software (Wächter and Biegler, 2005), using the linear solver *ma57* (HSL, 2013). In the *startup* simulations, 100 collocation points divided in 10 polynomials of order 10 are used in the propulsion phases and 144 collocation points divided in 12 polynomials of order 12 are used in the recovery phases to reduce mesh errors. In the *steady-state* simulations, 5 polynomials of order 20 in each phase are sufficient to minimize mesh errors. The mesh is weighted using a Legendre-Gauss-Lobatto polynomial to further reduce mesh errors, especially in the interface between phases. The first and second derivatives of the constraints and cost function are obtained through an automatic differentiation class using the forward mode written in MATLAB.

### 2.4. Simulations

The transient locomotion, representing the frequent activities of daily living where the wheelchair is accelerated from rest (Koontz et al., 2009), is referred to as *startup* simulation and is characterized by the sequence of phases propulsion-recovery-propulsion-recovery-propulsion, starting from rest, covering a predefined displacement, and at a given average speed. The steady-state locomotion, in turn, is referred to as *steady state*, represents locomotion over longer distances and is generated for one complete, periodic propulsion cycle at a predefined average speed.

The simulations are planned to investigate the effects of imposing a first-order linear reference model in the impedance control strategy and to assess the influence of changing the reference model parameters, *M*_*i*_ and *C*_*i*_, on user effort and energy consumption by the motor. The simulations are performed to cover a range of typical locomotion conditions, *steady state vs*. transient (*startup*), on a level surface *vs*. on a ramp.

The average speed is constrained to 0.9 m/s in both, *steady state* and *startup* simulations, while in the *startup* simulation a total displacement of 1.6 m is added as a constraint. For the simulations on a ramp, an inclination of η = 3° is adopted.

First, reference simulations of unassisted locomotion (motors turned off) are generated by solving the optimal control problem in Section 2.3 in all conditions: *steady state* on a level surface and on a ramp, and *startup* on a horizontal plane and on a ramp. The predictive simulations for assisted locomotion, with the impedance control loop in Figure 2, are generated using these reference simulations as initial guesses. When the convergence to a local minimum corresponding to clearly unrealistic patterns occurs, identified, for instance, by an unnaturally large push angle with an excessively posterior hand position on the pushrim in the beginning of the propulsion phase, the optimization is rerun with a different initial guess. This new initial guess corresponds to a predictive simulation generated for the same locomotion conditions and closest possible reference model parameters.

To investigate the impedance controller performance and its impacts on the wheelchair-user interaction we have applied a 50% reduction in the dynamic parameters of the reference model. This reduction represents a significant change in the parameters and therefore in the impedance, but it still keeps the user as the protagonist of the propulsion, who applies the largest part of the torque necessary to characterize the movement. We found through simulation that changes of 50% in the model parameters are sufficient to analyze its effects, while the maximum torque produced by the motor doesn't increase excessively.

In the assisted locomotion simulations, four combinations of the reference model mass and friction parameters are tested: 100% *M*_*i*_ and 100% *C*_*i*_ (nominal model, *M*_100_ − *C*_100_), 50% *M*_*i*_ and 100% *C*_*i*_ (*M*_50_ − *C*_100_), 100% *M*_*i*_ and 50% *C*_*i*_ (*M*_100_ − *C*_50_), and 50% *M*_*i*_ and 50% *C*_*i*_ (*M*_50_ − *C*_50_). The nominal simulation (*M*_100_ − *C*_100_) permits investigating the effects of imposing first-order linear dynamics in the impedance control loop and also to verify the disturbance rejection capability of this approach without changing overall system mass and friction properties. For instance, in locomotion on ramps, it is expected that the impedance control strategy provides the user the sensation of locomotion on a level surface. The other model parameter combinations allow for investigating the effects on locomotion performance of reducing apparent system mass and/or friction by 50%.

In summary, reference simulations are generated for locomotion without assistance, i.e., with the control loop turned off, for the four conditions, *steady state* and *startup* locomotion on a level surface and on a ramp. For each of these four locomotion conditions, four simulations of power-assisted locomotion are generated for the referred combinations of reference model parameters in the impedance control loop. Therefore, the total number of generated predictive simulations amounts to twenty.

## 3. Results

**Figure 3:**
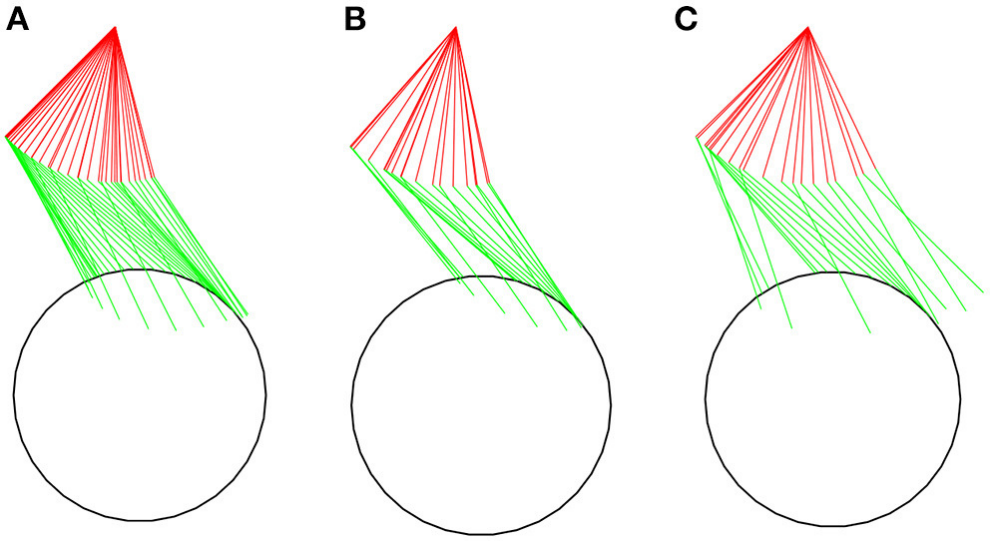
Stick figures representing snapshots of the predicted kinematics for unassisted locomotion on a level surface at an average speed of 0.9 m/s for a complete cycle in the *steady state* simulation **(A)**, for the first **(B)**, and second **(C)** complete cycles for the *startup* simulation.

The predicted patterns for the *steady state* simulation at 0.9*m*/*s* (Figure 3A) show overall agreement with kinematic, kinetic and spatiotemporal data reported in the literature. Tables 2, 3 present a comparison of parameters of the predicted patterns with data reported in Boninger et al. (2002) and Gil-Agudo et al. (2010) for different groups. The predicted cadence of 1.07*s*^−1^ is close to median values reported in Gil-Agudo et al. (2010). Predicted push time (0.395*s*) and recovery time (0.545*s*) are close to the reported in Gil-Agudo et al. (2010), leading to a ratio push time/recovery time of 0.73, which is lower than the reported in both papers, indicating a somewhat lower predicted duty cycle. In the case of Boninger et al. (2002), this difference can be attributed to the substantially larger reported push angles compared to the predicted push angle of 64.8°, which is close to the ones reported in Gil-Agudo et al. (2010). The push angle is influenced by factors such as adopted stroke patterns, wheelchair adjusting or trunk mobility, which may explain the differences in the reported push angles. The predicted maximal pushrim tangential force of 38.1*N* agrees well with the mean values reported in Boninger et al. (2002). The shoulder maximal flexion (7.6*N*.*m*) and extension moments (3.97*N*.*m*) agree well with the ones reported in Gil-Agudo et al. (2010). For the elbow, the maximal flexion moment (3.72*N*.*m*) shows good agreement, while the predicted maximal extension moment (2.3*N*.*m*) is larger in magnitude than the reported in Gil-Agudo et al. (2010), although not incompatible with expected patterns considering the large overall variability of the data reported in the literature.

**Table 2:** Comparison of kinematic, kinetic and spatiotemporal parameters of the *steady state* simulation at 0.9*m*/*s* to experimental data reported in Gil-Agudo et al. (2010) for locomotion at 3*km*/*h*≈0.83*m*/*s*.

	**Predicted**	**Gil-Agudo et al. (** **2010** **)** **median (interquartile range)**			
		**G1**	**G2**	**G3**	**G4**
Speed (m/s)	0.90	0.83	0.83	0.83	0.83
Max rim tangencial force (N)	38.1	-	-	-	-
Cadence (1/s)	1.07	0.9 (0.3)	1.1 (0.4)	1.1 (0.4)	1.2 (0.3)
Push time (s)	0.395	0.6 (0.3)	0.5 (0.1)	0.4 (0.5)	0.4 (0.1)
Recovery time (s)	0.545	0.5 (0.1)	0.5 (0.1)	0.5 (0.2)	0.5 (0.2)
Push/Recovery	0.73	1.3 (0.5)	1.1 (0.4)	0.8 (0.2)	0.8 (0.2)
Push angle (°)	64.8	62.5 (16.1)	58.6 (29.0)	64.5 (21.1)	57.5 (13.8)
Contact angle (°)	–110.1	–108.2 (18.5)	–111.3 (20.6)	–115.7 (17.5)	–110.4 (17.5)
Release angle (°)	–45.3	–51.3 (14.2)	–52.2 (16.1)	–54.0 (16.4)	-48.1 (13.4)
Max shoulder flex mom (N.m)	7.60	10.7 (4.6)	8.0 (3.2)	7.7 (4.8)	6.4 (4.6)
Max shoulder ext mom (N.m)	3.97	3.9 (4.5)	4.8 (2.3)	6.9 (2.5)	4.9 (2.6)
Max elbow flex mom (N.m)	3.72	4.6 (9.9)	4.9 (2.4)	6.0 (1.7)	4.6 (2.0)
Max elbow ext mom (N.m)	2.30	1.6 (1.3)	0.9 (1.0)	0.5 (0.9)	0.6 (0.9)

*Data is reported as median (interquartile range). The fifty-one people ere grouped by their level of spinal cord injury (SCI): C6 tetraplegia (G1), C7 tetraplegia (G2), high paraplegia (G3), and low paraplegia (G4). “-” represents data not reported in the paper*.

**Table 3:** Comparison of kinematic, kinetic and spatiotemporal parameters of the *steady state* simulation at 0.9*m*/*s* to experimental data reported in Boninger et al. (2002) for locomotion at 0.9*m*/*s*.

	**Predicted**	**Boninger et al. (** **2002** **)** **mean (standard deviation)**			
		**ARC**	**SC**	**SLOP**	**DLOP**
Speed (m/s)	0.90	0.9	0.9	0.9	0.9
Max rim tangencial force (N)	38.1	45.8 (22.1)	38.3 (20.6)	47.0 (19.4)	65.8 (26.6)
Cadence (1/s)	1.07	1.13 (0.18)	0.88 (0.08)	1.03 (0.14)	0.81 (0.13)
Push time (s)	0.395	-	-	-	-
Recovery time (s)	0.545	-	-	-	-
Push/Recovery	0.73	1.12 (0.19)	1.21 (0.31)	1.03 (0.28)	0.78 (0.18)
Push angle (°)	64.8	94.4 (24.1)	114.0 (13.7)	91.7 (13.8)	110.0 (13.0)
Contact angle (°)	-110.1	-	-	-	-
Release angle (°)	-45.3	-	-	-	-
Max shoulder flex mom (N.m)	7.60	-	-	-	-
Max shoulder ext mom (N.m)	3.97	-	-	-	-
Max elbow flex mom (N.m)	3.72	-	-	-	-
Max elbow ext mom (N.m)	2.30	-	-	-	-

*Data is reported as mean (standard deviation). The thirty-eight individuals with paraplegia are grouped according to their selected stroke pattern in the recovery phase: semicircular (SC), SLOP, DLOP, and arcing (ARC). “-” represents data not reported in the paper*.

**Figure 4:**
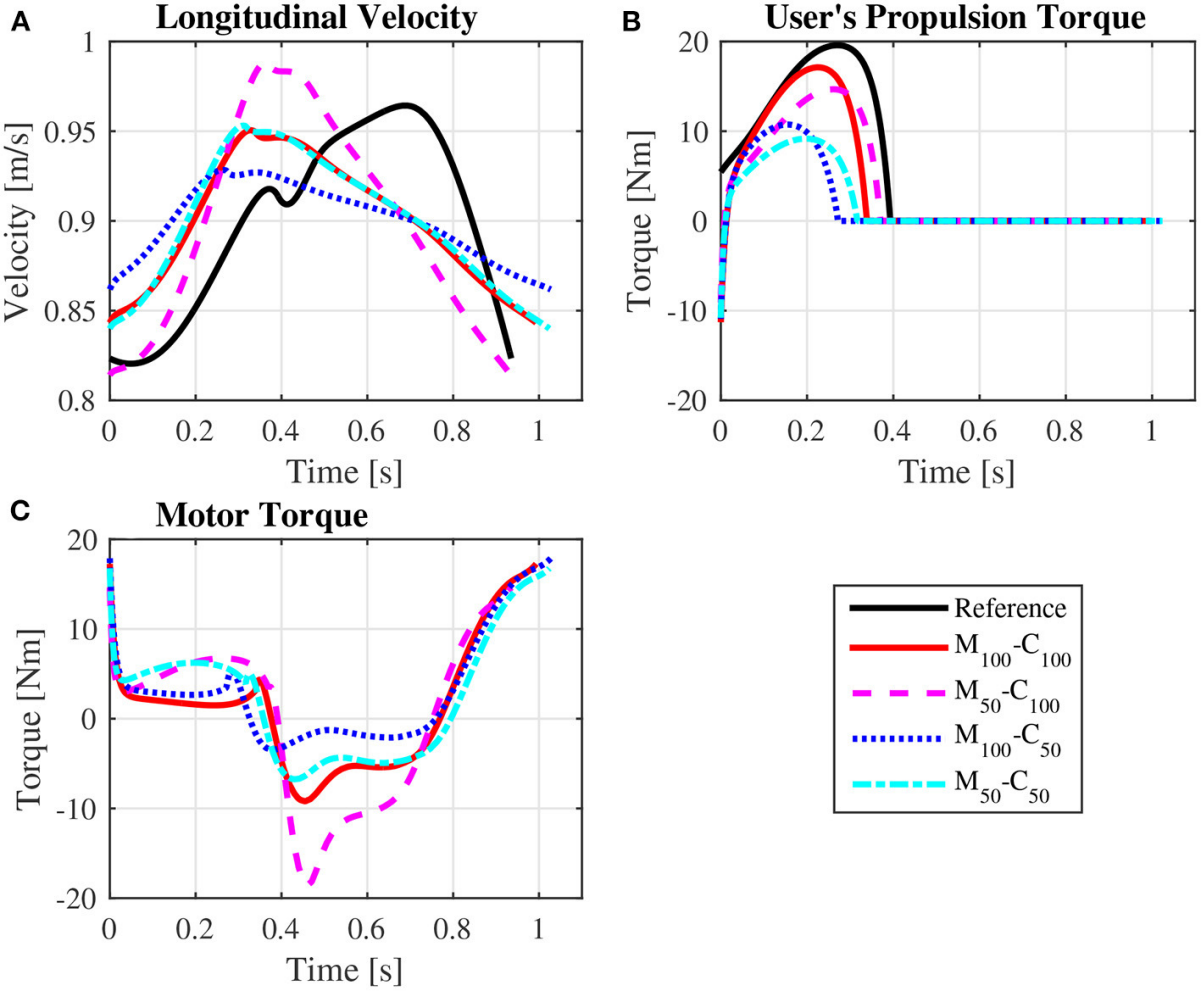
Predicted wheelchair speed profile **(A)**, bilateral propulsion torque applied by the user **(B)**, and bilateral torque applied by the motors on the rear wheels of the wheelchair **(C)** for *steady state* locomotion on a level surface at an average speed of 0.9 m/s for the reference unassisted condition (Reference), and for the assisted locomotion with 100% of reference model parameters *M*_*i*_ and *C*_*i*_ (*M*_100_ − *C*_100_), reduction of 50% in *M*_*i*_ (*M*_50_ − *C*_100_), reduction of 50% in *C*_*i*_ (*M*_100_ − *C*_50_), and reduction of 50% in *M*_*i*_ and *C*_*i*_ (*M*_50_ − *C*_50_).

**Figure 5:**
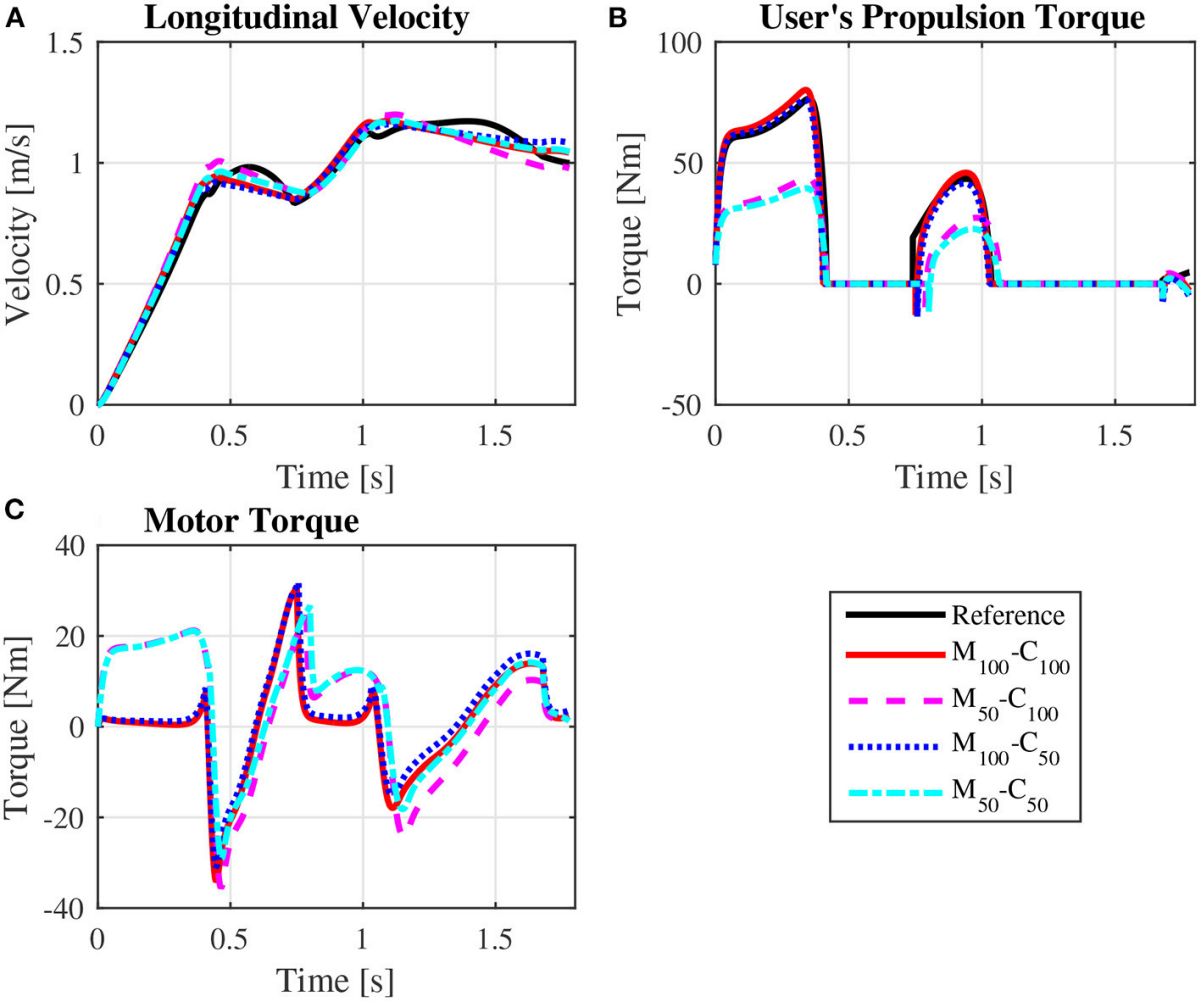
Predicted wheelchair speed profile **(A)**, bilateral propulsion torque applied by the user **(B)**, and bilateral torque applied by the motors on the rear wheels of the wheelchair **(C)** along the sequence of phases propulsion-recovery-propulsion-recovery-propulsion in the startup locomotion on a level surface at an average speed of 0.9 m/s for the reference unassisted condition (Reference), and for the assisted locomotion with 100% of reference model parameters Mi and Ci (*M*_100_ − *C*_100_), reduction of 50% in Mi (*M*_50_ − *C*_100_), reduction of 50% in Ci (*M*_100_ − *C*_50_), and reduction of 50% in Mi and Ci (*M*_50_ − *C*_50_).

The predicted wheelchair speed profile for the reference simulation on a level surface for *steady state* locomotion (solid black line in Figure 4A) shows the expected acceleration in the propulsion phase, in which the system kinetic energy increases due to positive work exerted by the hands on the pushrim. In the recovery phase, starting at *t* = 0.395*s*, the center of mass of the whole user-wheelchair system decelerates under the action of the rolling resistance force. However, the wheelchair speed profile does show an acceleration in the first half of the recovery phase, a behavior caused by the dynamic effect associated with the backwards motion of the upper limbs. The same effect occurs in the two recovery phases of the unassisted reference *startup* simulation for locomotion on a level surface (Figures 3B,C, and solid black line in Figure 5A).

**Figure 6:**
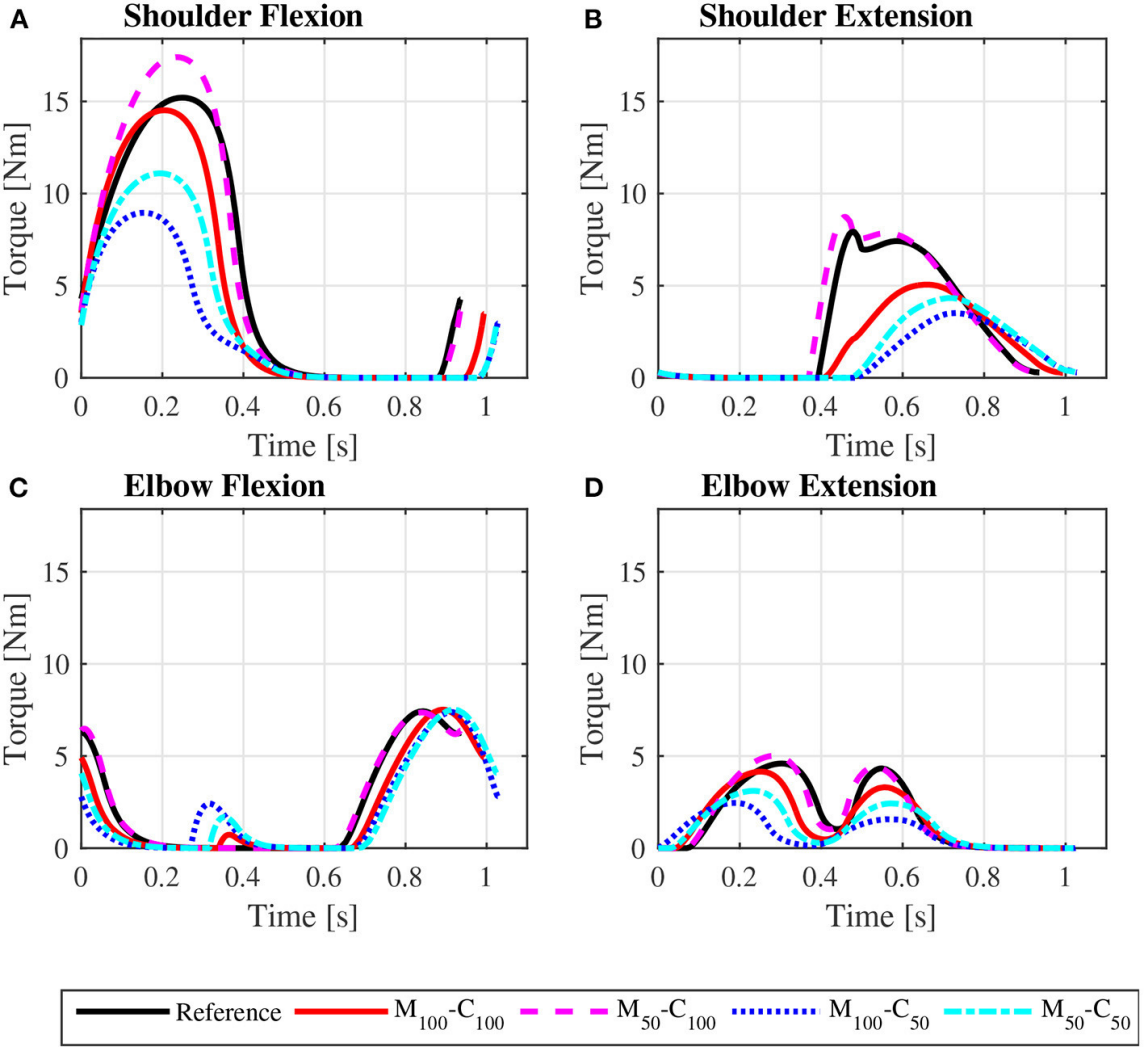
Predicted bilateral shoulder flexion **(A)**, shoulder extension **(B)**, elbow flexion **(C)**, and elbow extension **(D)** torque profiles along a complete cycle for steady state locomotion on a level surface at an average speed of 0.9 m/s for the reference unassisted condition (Reference), and for the assisted locomotion with 100% of reference model parameters Mi and Ci (*M*_100_ − *C*_100_), reduction of 50% in Mi (*M*_50_ − *C*_100_), reduction of 50% in Ci (*M*_100_ − *C*_50_), and reduction of 50% in Mi and Ci (*M*_50_ − *C*_50_).

**Figure 7:**
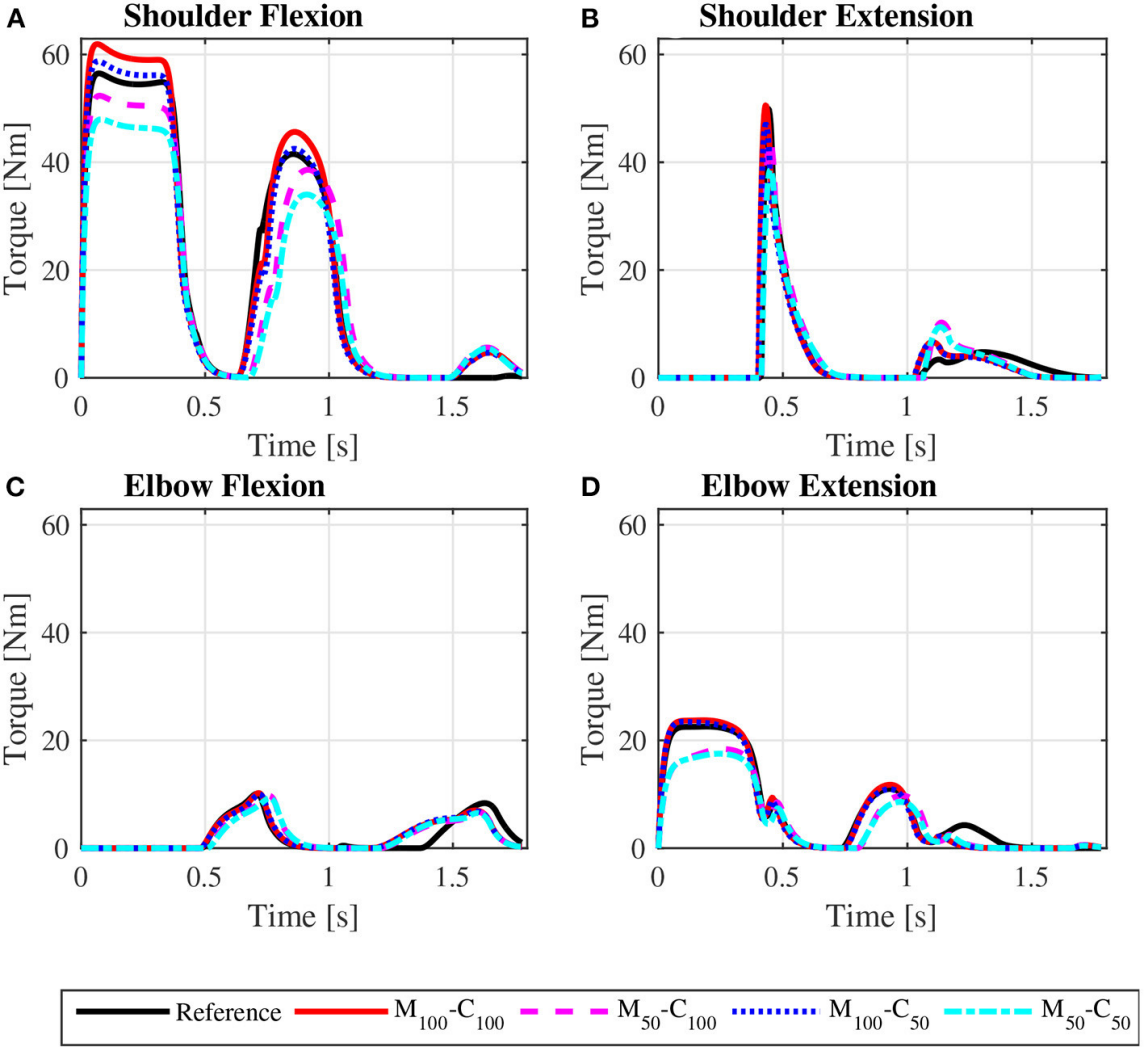
Predicted bilateral shoulder flexion **(A)**, shoulder extension **(B)**, elbow flexion **(C)**, and elbow extension **(D)** torque profiles along the sequence of phases propulsion-recovery-propulsion-recovery-propulsion in the startup locomotion on a level surface at an average speed of 0.9 m/s for the reference unassisted condition (Reference), and for the assisted locomotion with 100% of reference model parameters Mi and Ci (*M*_100_ − *C*_100_), reduction of 50% in Mi (*M*_50_ − *C*_100_), reduction of 50% in Ci (*M*_100_ − *C*_50_), and reduction of 50% in Mi and Ci (*M*_50_ − *C*_50_).

**Figure 8:**
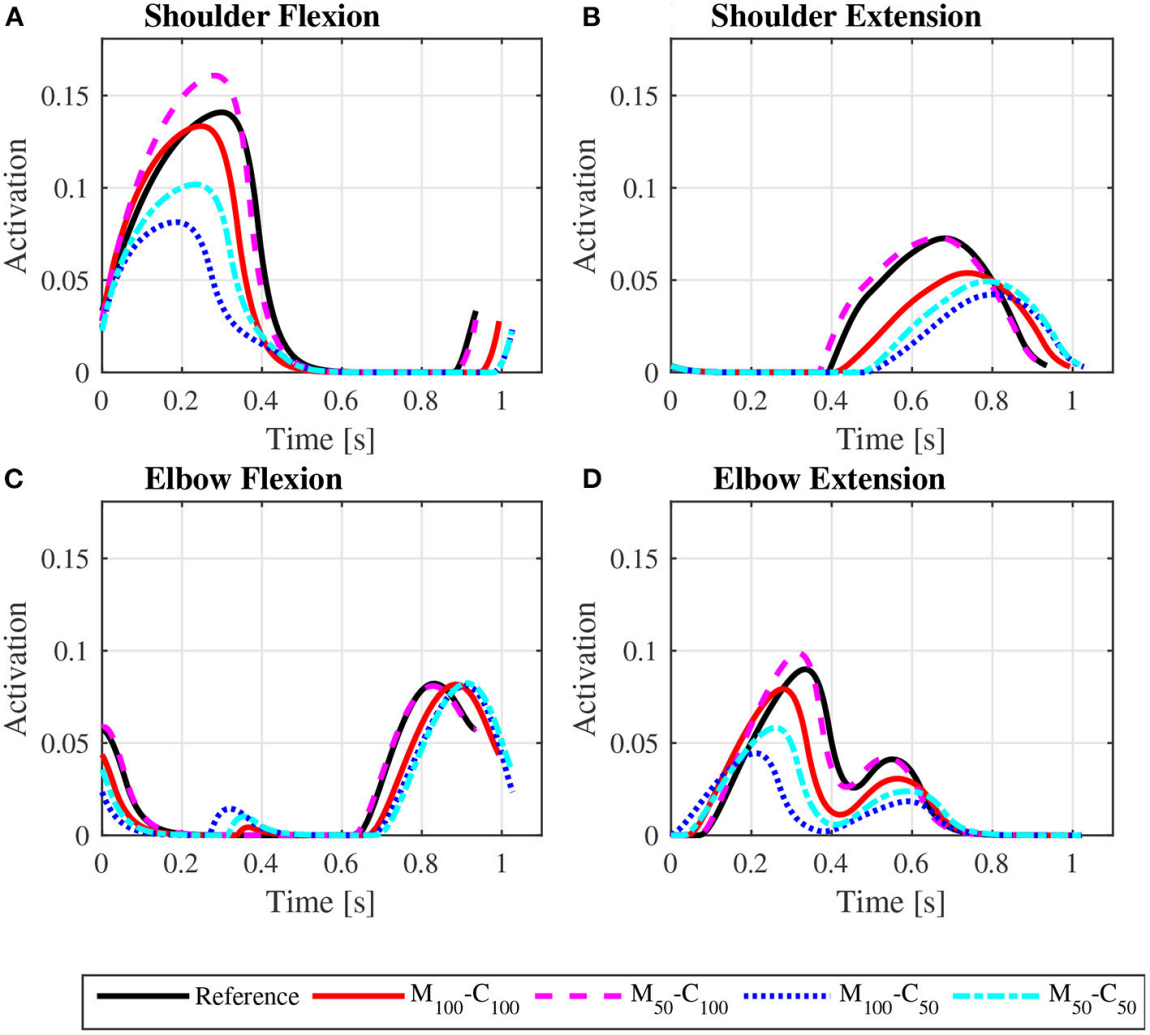
Predicted shoulder flexion **(A)**, shoulder extension **(B)**, elbow flexion **(C)** and elbow extension **(D)** activation profiles along a complete cycle for steady state locomotion on a level surface at an average speed of 0.9 m/s for the reference unassisted condition (Reference), and for the assisted locomotion with 100% of reference model parameters Mi and Ci (*M*_100_ − *C*_100_), reduction of 50% in Mi (*M*_50_ − *C*_100_), reduction of 50% in Ci (*M*_100_ − *C*_50_), and reduction of 50% in Mi and Ci (*M*_50_ − *C*_50_).

**Figure 9:**
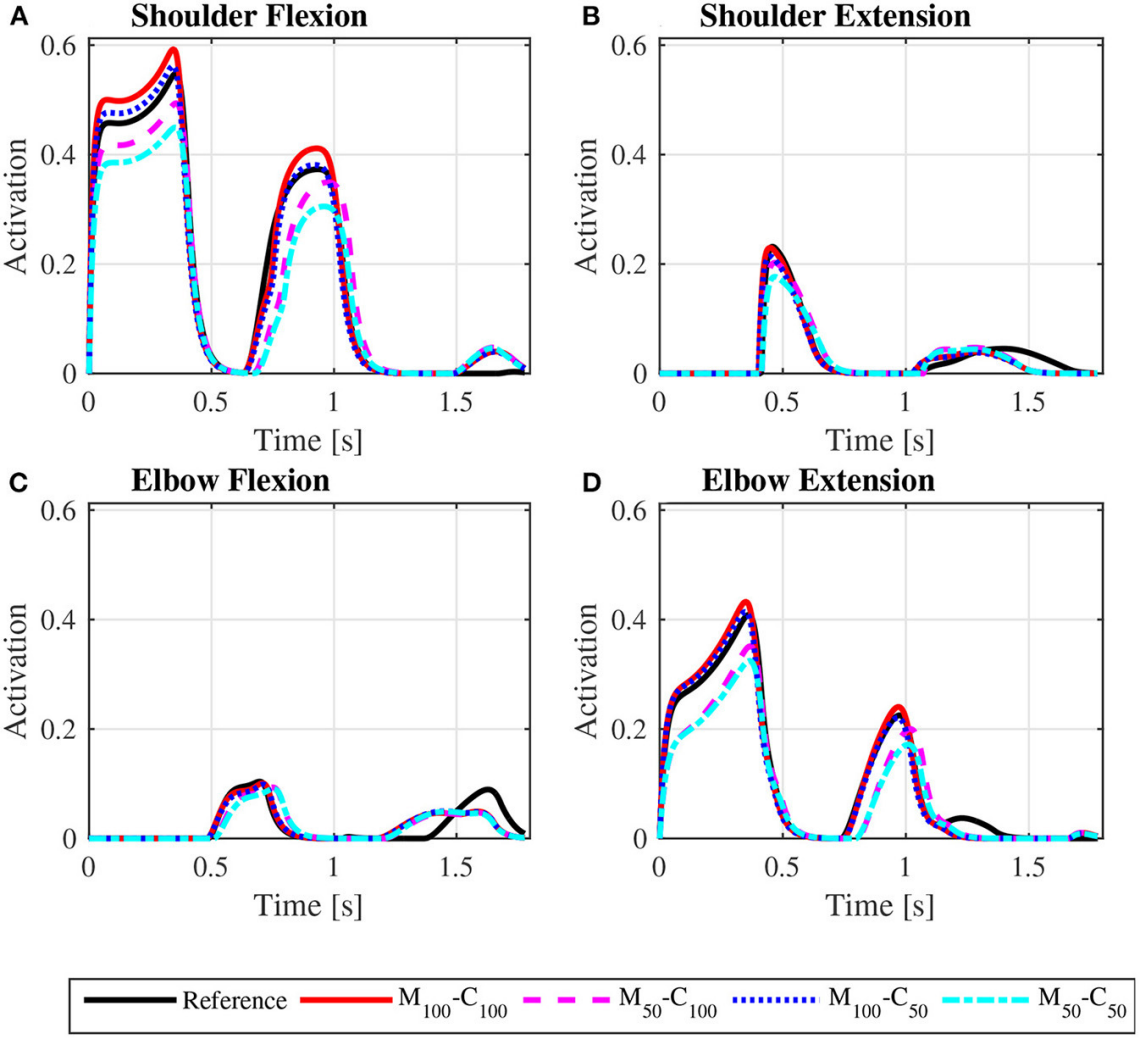
Predicted shoulder flexion **(A)**, shoulder extension **(B)**, elbow flexion **(C)**, and elbow extension **(D)** activation profiles along the sequence of phases propulsion-recovery-propulsion-recovery-propulsion in the *startup* locomotion on a level surface at an average speed of 0.9 m/s for the reference unassisted condition (Reference), and for the assisted locomotion with 100% of reference model parameters *M*_*i*_ and *C*_*i*_ (*M*_100_ − *C*_100_), reduction of 50% in *M*_*i*_ (*M*_50_ − *C*_100_), reduction of 50% in *C*_*i*_ (*M*_100_ − *C*_50_), and reduction of 50% in *M*_*i*_ and *C*_*i*_ (*M*_50_ − *C*_50_).

The predicted joint torque profiles for the reference unassisted locomotion on a level surface (Figure 6 for *steady state* and Figure 7 for *startup*) show that the largest torques are applied by the shoulder in flexion during the propulsion phases, with shoulder flexion activations achieving peaks of about 0.14 in *steady state* (Figure 8A) and 0.55 in *startup* (Figure 9A). The activation profiles (Figures 8, 9) lead to similar joint torque profiles (Figures 6, 7), modulated by the intrinsic muscle properties. Note, for instance, that the elbow extension torque peaks in Figure 6D, in the end of the propulsion phase (t≈0.35 s) and in the beginning of the recovery phase (t≈0.55 s), have similar magnitudes, in spite of the different corresponding elbow activation peaks shown in Figure 8D. This difference is due to the effect of the force-velocity relationship, which reduces force generation capacity of the elbow extensor muscles during the concentric contraction at larger rates close to full elbow extension in the end of the propulsion phase, leading to a larger necessary activation to generate the same torque.

The identification of the first-order linear model used as reference in the impedance control strategy resulted in a dynamic friction parameter of *C*_*i*_ = 14.88 N.s/m and a mass parameter of *M*_*i*_ = 92.50 kg, which is consistent with system overall mass (wheelchair + user).

Note that the typical speed profile in the recovery phase of the unassisted reference locomotion vanishes in the assisted locomotion simulations, which are characterized mostly by a monotonic speed decrease along the recovery phase (Figures 4A, 5A). Since the reference model in the impedance control loop in Figure 2 corresponds to a block of mass *M*_*i*_ under the effect of a viscous damping *C*_*i*_, the reference velocity *v*_*r*_ decreases exponentially in the recovery phase in which τ_*p*_ = 0. In this condition, the control loop ends up suppressing the wheelchair acceleration as the upper extremity is moved backwards by applying a negative motor torque τ_*m*_. This behavior occurs particularly in the first half of the recovery phase, as clearly shown in Figure 4C (from *t* ≈ 0.4*s* to *t* ≈ 0.8*s*) and Figure 5C (from *t* ≈ 0.4*s* to *t* ≈ 0.6*s* and from *t* ≈ 1.1*s* to *t* ≈ 1.4*s*). This explains to a great extent why motor effort is far from null in the assisted, nominal simulations with 100% of *M*_*i*_ and 100% of *C*_*i*_ (*W*_*m*_ for condition *M*_100_ − *C*_100_ in Table 4).

**Table 4:** Predicted user effort *W*_*p*_ (Equation 9) and motor effort *W*_*m*_ (Equation 10) for all simulated conditions.

**Condition**	***W*_*p*_ [10^−1^*s*]**	***W*_*m*_ [*N*^2^*m*^2^*s*]**
**Level/** ***steady state***		
Reference	0.107	-
*M*_100_ − *C*_100_	0.079	48.92
*M*_50_ − *C*_100_	0.124	76.14
*M*_100_ − *C*_50_	0.035	46.46
*M*_50_ − *C*_50_	0.051	52.11
**Ramp/** ***steady state***		
Reference	0.733	-
*M*_100_ − *C*_100_	0.164	183.95
*M*_50_ − *C*_100_	0.237	155.12
*M*_100_ − *C*_50_	0.084	244.66
*M*_50_ − *C*_50_	0.116	213.63
**Level/** ***startup***		
Reference	2.00	-
*M*_100_ − *C*_100_	2.22	206.10
*M*_50_ − *C*_100_	1.57	380.02
*M*_100_ − *C*_50_	1.99	202.57
*M*_50_ − *C*_50_	1.30	339.92
**Ramp/** ***startup***		
Reference	4.39	-
*M*_100_ − *C*_100_	2.75	410.77
*M*_50_ − *C*_100_	2.05	611.11
*M*_100_ − *C*_50_	2.48	477.74
*M*_50_ − *C*_50_	1.71	687.29

The results for assisted *steady state* locomotion on a level surface show that reducing the apparent coefficient of friction (*M*_100_ − *C*_50_ and *M*_50_ − *C*_50_) is effective in decreasing user's wheel torque (Figure 4B) as well as shoulder and elbow torques (Figure 6) and the corresponding activations (Figure 8). This explains the reduction in user's effort (*W*_*p*_) shown in Table 4 for these conditions. For example, the maximum shoulder flexion torque changes from approximately 15*N*.*m* to 12*N*.*m* in the *M*_50_ − *C*_50_ condition and to 9*N*.*m* in the *M*_100_ − *C*_50_ condition (Figure 6A). A reduction in apparent mass alone, on the contrary, has a relatively small effect on the user's propulsion torque profile (*M*_50_ − *C*_100_ in Figure 4B). In fact, the *M*_50_ − *C*_100_ condition leads to increases in both user and motor effort (Table 4), reflecting larger shoulder flexion torque and activation compared to the reference unassisted simulation (Figures 6A, 8A), even in the presence of larger motor torque magnitudes (Figure 4C). This shows that an isolated decrease in apparent system mass in the investigated impedance control strategy is detrimental to performance in *steady state* locomotion on a level surface.

The scenario is different for the assisted *startup* locomotion on a level surface. The reduction in apparent viscous friction alone (*M*_100_ − *C*_50_) has little influence on user propulsion torque profile (Figure 5B), joint torques (Figure 7) and activations (Figure 9) compared to the unassisted condition, leading to similar user's effort *W*_*p*_ in Table 4. The reduction in apparent mass, instead, cuts user propulsion torque by half (*M*_50_ − *C*_100_ and *M*_50_ − *C*_50_ in Figure 5B), substantially decreasing shoulder flexion and elbow extension torques (Figures 7A,D) and the corresponding activations (Figures 9A,D) in the propulsion phases. This is achieved through the assistance provided by a larger motor torque in the propulsion phases (Figure 5C, from *t* = 0 to *t* ≈ 0.4*s* and from *t* ≈ 0.8*s* to *t* ≈ 1.05*s*).

**Figure 10:**
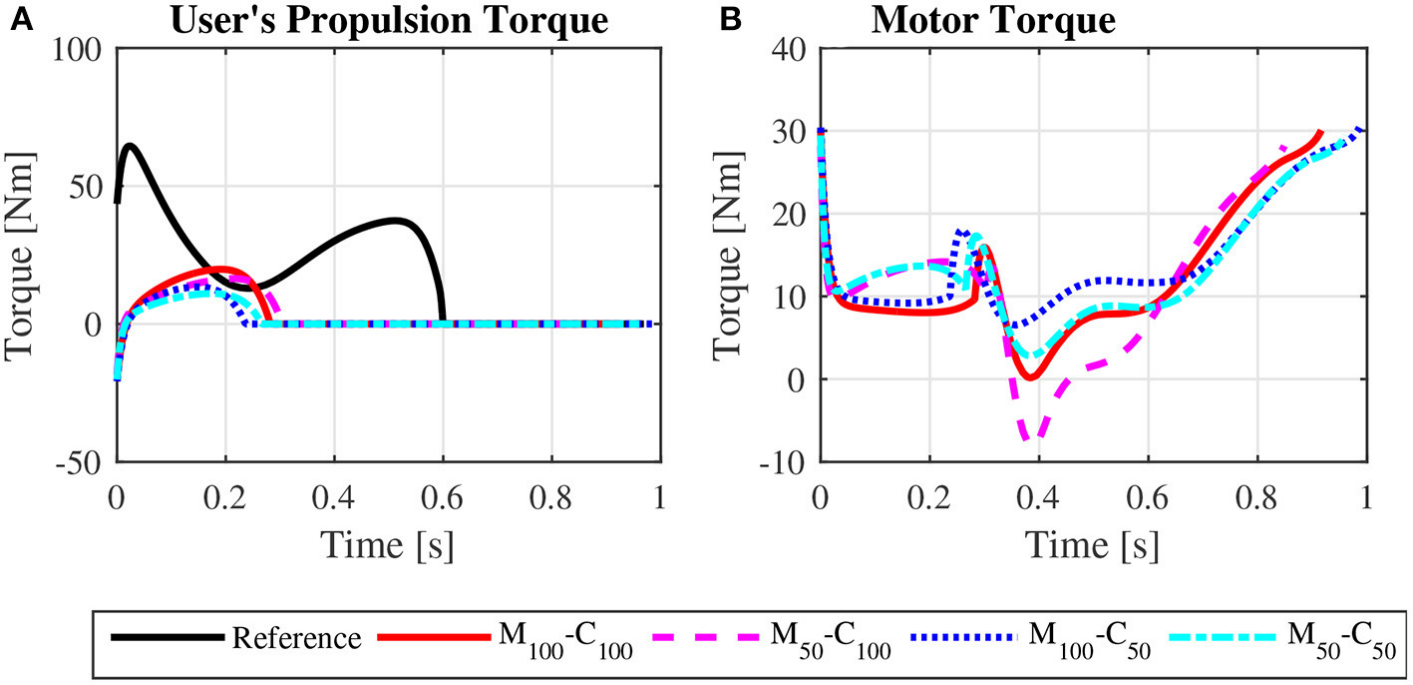
Bilateral propulsion torque applied by the user **(A)** and bilateral torque applied by the motors on the rear wheels of the wheelchair **(B)** for *steady state* locomotion on a 3° ramp at an average speed of 0.9 m/s for the reference unassisted condition (Reference), and for the assisted locomotion with 100% of reference model parameters *M*_*i*_ and *C*_*i*_ (*M*_100_ − *C*_100_), reduction of 50% in *M*_*i*_ (*M*_50_ − *C*_100_), reduction of 50% in *C*_*i*_ (*M*_100_ − *C*_50_), and reduction of 50% in *M*_*i*_ and *C*_*i*_ (*M*_50_ − *C*_50_).

**Figure 11:**
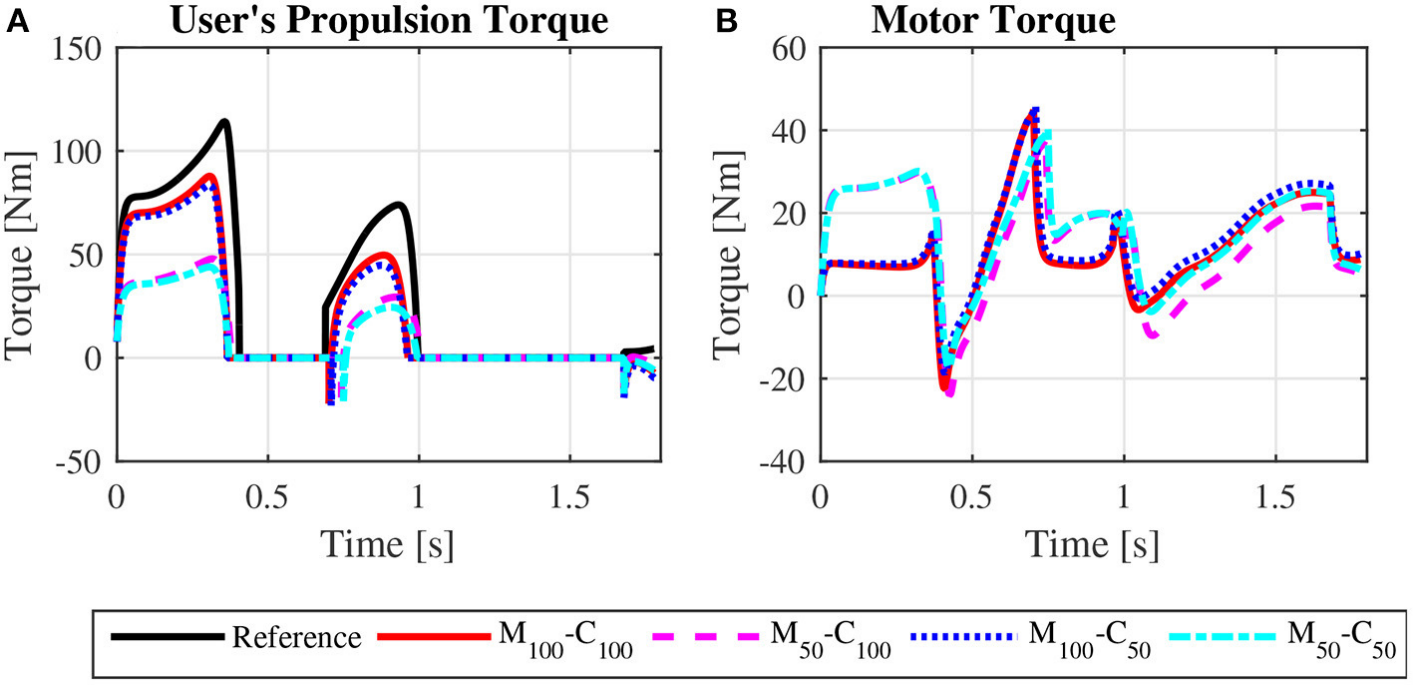
Bilateral propulsion torque applied by the user **(A)** and bilateral torque applied by the motors on the rear wheels of the wheelchair **(B)** for *startup* locomotion on a 3° ramp at an average speed of 0.9 m/s for the reference unassisted condition (Reference), and for the assisted locomotion with 100% of reference model parameters *M*_*i*_ and *C*_*i*_ (*M*_100_ − *C*_100_), reduction of 50% in *M*_*i*_ (*M*_50_ − *C*_100_), reduction of 50% in *C*_*i*_ (*M*_100_ − *C*_50_), and reduction of 50% in *M*_*i*_ and *C*_*i*_ (*M*_50_ − *C*_50_).

The user propulsion torque profiles for the four reference model parameter combinations in assisted *steady state* locomotion on a 3° ramp, depicted in Figure 10A, are much lower than those for the reference simulation and similar to those predicted for locomotion on a level surface, Figure 4B. The same occurs for the assisted *startup* locomotion, as shown in Figure 11A, compared to results for the reference *startup* locomotion on a level surface in Figure 5B. The predicted muscle activation profiles for shoulder and elbow extensors and flexors for the assisted locomotion on the ramp (Supplementary Figure S4 for *steady state*, Supplementary Figure S7 for *startup*) are similar to those predicted for locomotion on a level surface (Figure 8 for *steady state*, Figure 9 for *startup*). Note that user effort values *W*_*p*_ reported in Table 4 for the assisted locomotion on a ramp for both, *steady state* and *startup*, are much lower than those for the unassisted reference simulations of locomotion on the ramp. This compensation of gravity in the assisted locomotion on ramps is ensured by an offset of motor torque profiles in *steady state*, Figure 10B compared to Figure 4C, and in *startup*, Figure 11B compared to Figure 5C. This motor action is associated to a substantial motor effort *W*_*m*_ (Table 4) and consequent large energy consumption from the batteries.

## 4. Discussion

The predicted patterns for the reference *steady state* locomotion at 0.9*m*/*s* shows overall agreement with data reported in the literature (Boninger et al., 2002; Gil-Agudo et al., 2010), with the isolated observed differences compatible with the typical variability in reported wheelchair propulsion patterns in the literature. This indicates that the employed model and optimal control approach are able to generate realistic wheelchair locomotion patterns.

The results for the assisted locomotion indicate that the studied impedance control strategy can effectively impose the reference dynamics, despite the nonlinear dynamic nature of the wheelchair-user system. This provides a natural way of adjusting assistance intensity by manipulation of mass and damping parameters in the reference model and leads to automatic compensation of external disturbances such as the effect of gravity on slopes or changes in rolling resistance force in different pavements or rough terrain. These constitute highly desirable characteristics of the impedance control strategy. In fact, the predicted profiles for locomotion on a 3° ramp show that the impedance control strategy effectively rejects the disturbance due to the weight component in the direction of motion, so that the required user joint torques, muscle activation profiles and muscle effort are similar to those observed during locomotion on a level surface. This effectively gives to the user the impression of propelling the wheelchair on a level surface during upwards locomotion on a ramp.

Despite the benefits of the investigated control strategy, the simulations also reveal that imposing a first-order reference model in the impedance control loop might cause undesired side effects. The commonly used reference model seems adequate for a situation in which a caregiver pulls the wheelchair-user system. However, when the user self-propels the wheelchair, the dynamics of the movement becomes markedly nonlinear, mainly in the recovery phase when the motion of the arms changes the system dynamics in a way not captured by the first-order impedance control reference model that has the tangential force on the pushrim and the wheelchair wheel angular speed as the only inputs. The result is that the impedance control treats these system nonlinearities as disturbances and tries to reject them. This rejection leads to unnecessary energy dissipation and battery power consumption to produce unnatural movement.

This waste of energy is evident in the predicted nominal simulations with 100% of *M*_*i*_ and 100% of *C*_*i*_. One would expect this parameter configuration would lead to low control effort, as the reference model approximates the dynamics of the original user-wheelchair system. In spite of that, Table 4 reports large motor effort for the condition *M*_100_ − *C*_100_ during locomotion on a level surface. This effect is associated to a great extent with large motor breaking torques in the first half of the recovery phase to suppress the forward acceleration of the wheelchair as arms are accelerated backwards (Figures 4C, 5C).

Different strategies can mitigate these undesired effects. One possibility is to turn off assistance in the recovery phase or impose different dynamics for each phase. Another possible strategy is to formulate an impedance control with a proportional-derivative control law based solely on the measurement of the wheelchair velocity. This strategy dispenses the measurement of pushrim tangential forces, but, since it does not impose a reference dynamics, it does not compensate for external disturbances such as slopes. A third possible strategy is to use a reference model that can also capture the nonlinear dynamics associated with the swinging of the arms.

The opposing effects of mass and viscous damping parameters on *steady state vs. startup* locomotion can be attributed to their different nature. The *startup* locomotion represents transient maneuvers, where inertial forces are more important due to larger accelerations. In *steady state* locomotion, instead, accelerations are lower and average speed is greater, leading to the predominant effect of viscous damping over mass. In daily wheelchair use, which encompasses transient as well as steady-state locomotion, an isolated reduction in *C*_*i*_ could lead to overall improvement in terms of user effort, but only a concomitant reduction in both apparent parameters, friction and mass, can lead to a substantial user effort decrease in all investigated conditions, as indicated in Table 4. Thus, the reduction in both parameters seems to be the most indicated choice when adjusting assistance intensity level.

The employed model in this study allowed for a realistic investigation of wheelchair propulsion, while ensuring continuity and computational efficiency, important features for solving optimal control problems. The model appropriately represents the two phases of the locomotion cycle, the primary dynamic effects related to the motion of the upper limbs, and the intrinsic muscle properties. Nevertheless, a set of limitations must be mentioned. Adopting a muscle model formulated on the joint level should be sufficient to characterize muscle system capacity in assessing overall system performance, but it does not consider individual muscles, which may be important in studies concerned with joint loads and muscle coordination. Assuming the shoulder joint fixed to the wheelchair dispenses the use of complex and computationally costly trunk models but neglects the possible forward projection of the trunk during propulsion, better representing populations with a lower trunk mobility. A 2D model represents appropriately the upper limb dynamics and its interaction with the power-assisted wheelchair as they occur predominantly in the sagittal plane, but do not represent, for instance, the relevant shoulder abduction in the beginning of the propulsion phase. Finally, the adoption of force-length and force-velocity relationships from data obtained by Brown (2018) for an elite wheelchair basketball athlete leads to a model with larger force capacity than the average wheelchair user population, but this should have limited impact on overall predicted patterns as the simulations are submaximal, except for the reference *startup* simulation on a ramp at the relatively large average speed of 0.9*m*/*s*, that reaches a peak shoulder flexion activation of nearly 0.9. These limitations will be addressed in future studies as trunk and upper extremity models become more available and computationally tractable.

## 5. Conclusion

This work investigates the benefits and drawbacks of implementing an impedance control strategy in assisted wheelchair locomotion, adopting a first-order linear mass-damper model as reference dynamics, a recurrent choice in the literature. A realistic physiological model of the user's musculoskeletal system and its interaction with the power-assisted wheelchair was developed and used in predictive simulations of steady-state locomotion and of starting up from rest, representing common transient maneuvers in activities of daily living. The model allowed for taking the dynamic effects of arm motion, the intrinsic muscle properties, and the varying system dynamics in the propulsion and recovery phases into account.

The results confirm the advantages of the studied impedance control strategy, including automatic compensation of gravity forces in inclined terrains and the possibility of naturally adjusting assistance by manipulation of physical parameters such as mass and damping. An important finding, however, is that assuming a mass-damper model as reference in the impedance control loop leads to unnecessary braking in the recovery phase, since the natural forward motion of the wheelchair as the arms retreat is suppressed by the control loop as a disturbance, leading to waste of energy and performance degradation. Proposed solutions to this behavior include turning off motor assistance in the recovery phase, switching reference models depending on the locomotion phase, or incorporating the upper extremity nonlinear dynamics into the impedance control reference model. These strategies will be investigated in future studies using the developed predictive simulation approach.

Regarding the effects of manipulating the reference model parameters, the results reveal that reducing apparent mass effectively decreases user effort in transient maneuvers but is detrimental to performance in steady-state locomotion, where inertial forces are less important. On the contrary, reducing the damping parameter is advantageous in steady-state locomotion but affects only marginally the performance in transient maneuvers. A concomitant reduction in both parameters, apparent damping and mass, is able to substantially decrease user effort in all investigated conditions, constituting, therefore, the most indicated strategy for daily wheelchair use.

We expect the reported findings as well as the proposed simulation framework will provide guidance to the development of better control strategies for power-assisted wheelchairs. Future studies will employ more complete biomechanical models of the upper limbs and incorporate braking and locomotion in curves. Experimental studies with a prototype of a power-assisted wheelchair are planned for validation and testing.

## Data Availability Statement

The raw data supporting the conclusions of this article will be made available by the authors, without undue reservation.

## Author Contributions

MA developed and implemented the model. FL developed and implemented the control system. VC designed and ran the simulations. All authors have designed the study and elaborated the manuscript.

## Funding

This study was partially funded by the Coordenação de Aperfeiçoamento de Pessoal de Nível Superior—Brasil (CAPES)-Finance Code 001.

## Conflict of Interest

The authors declare that the research was conducted in the absence of any commercial or financial relationships that could be construed as a potential conflict of interest.

## Publisher's Note

All claims expressed in this article are solely those of the authors and do not necessarily represent those of their affiliated organizations, or those of the publisher, the editors and the reviewers. Any product that may be evaluated in this article, or claim that may be made by its manufacturer, is not guaranteed or endorsed by the publisher.
