# Supplementary material for: Performance of Impedance Control-Based Strategies in Power-Assisted Wheelchairs: A Predictive Simulation Study
Source: Front Neurorobot. 2022 Mar 4;16:805835. doi: 10.3389/fnbot.2022.805835 (PMC8930826; doi:10.3389/fnbot.2022.805835)
Supplement: Supplementary file 1 [file Data_Sheet_1.PDF]

![frontiers]

Supplementary Material

1 FIGURES

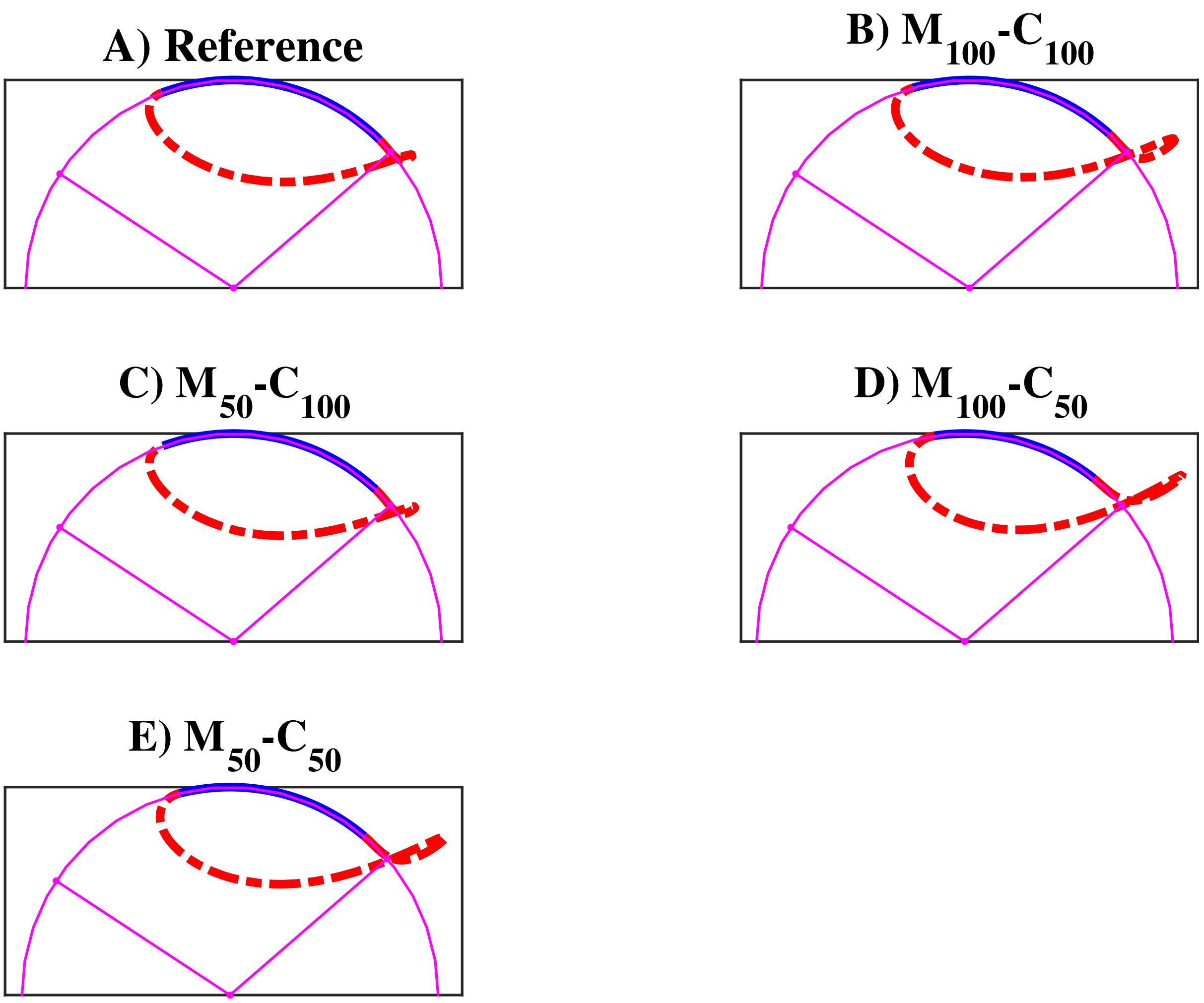

Figure S1. Predicted hand patterns for the reference unassisted condition (Reference) **(A)**, for the assisted locomotion with 100% of reference model parameters M_i and C_i ($M_{100} - C_{100}$) **(B)**, reduction of 50% in M_i ($M_{50} - C_{100}$) **(C)**, reduction of 50% in C_i ($M_{100} - C_{50}$) **(D)**, and reduction of 50% in M_i and C_i ($M_{50} - C_{50}$) **(E)** along a complete cycle for *steady state* locomotion on a level surface at an average speed of 0.9 m/s.

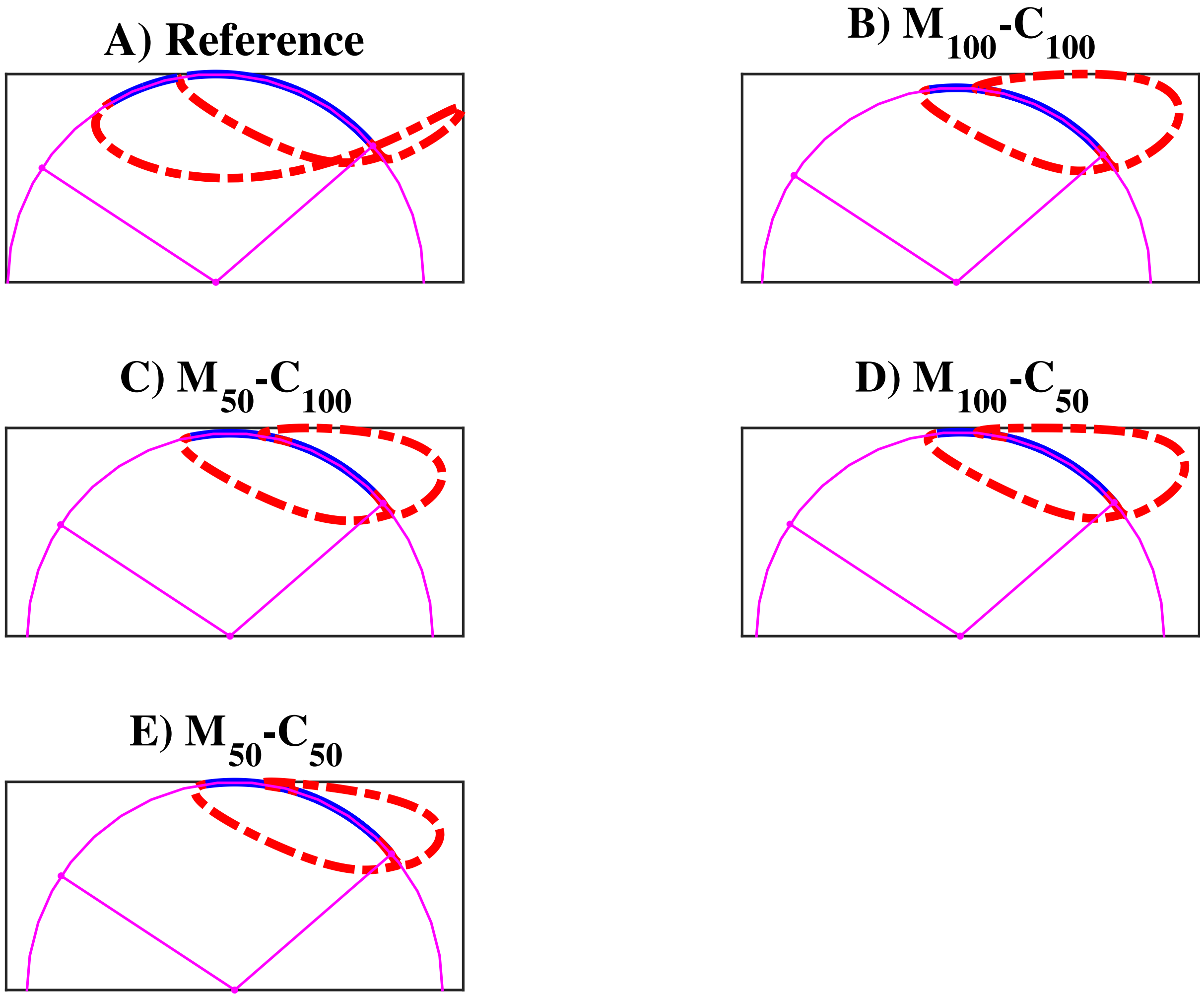

Figure S2. Predicted hand patterns for the reference unassisted condition (Reference) **(A)**, for the assisted locomotion with 100% of reference model parameters M_i and C_i ($M_{100} - C_{100}$) **(B)**, reduction of 50% in M_i ($M_{50} - C_{100}$) **(C)**, reduction of 50% in C_i ($M_{100} - C_{50}$) **(D)**, and reduction of 50% in M_i and C_i ($M_{50} - C_{50}$) **(E)** along the sequence of phases propulsion-recovery-propulsion-recovery-propulsion in the *startup* locomotion on a level surface at an average speed of 0.9 m/s.

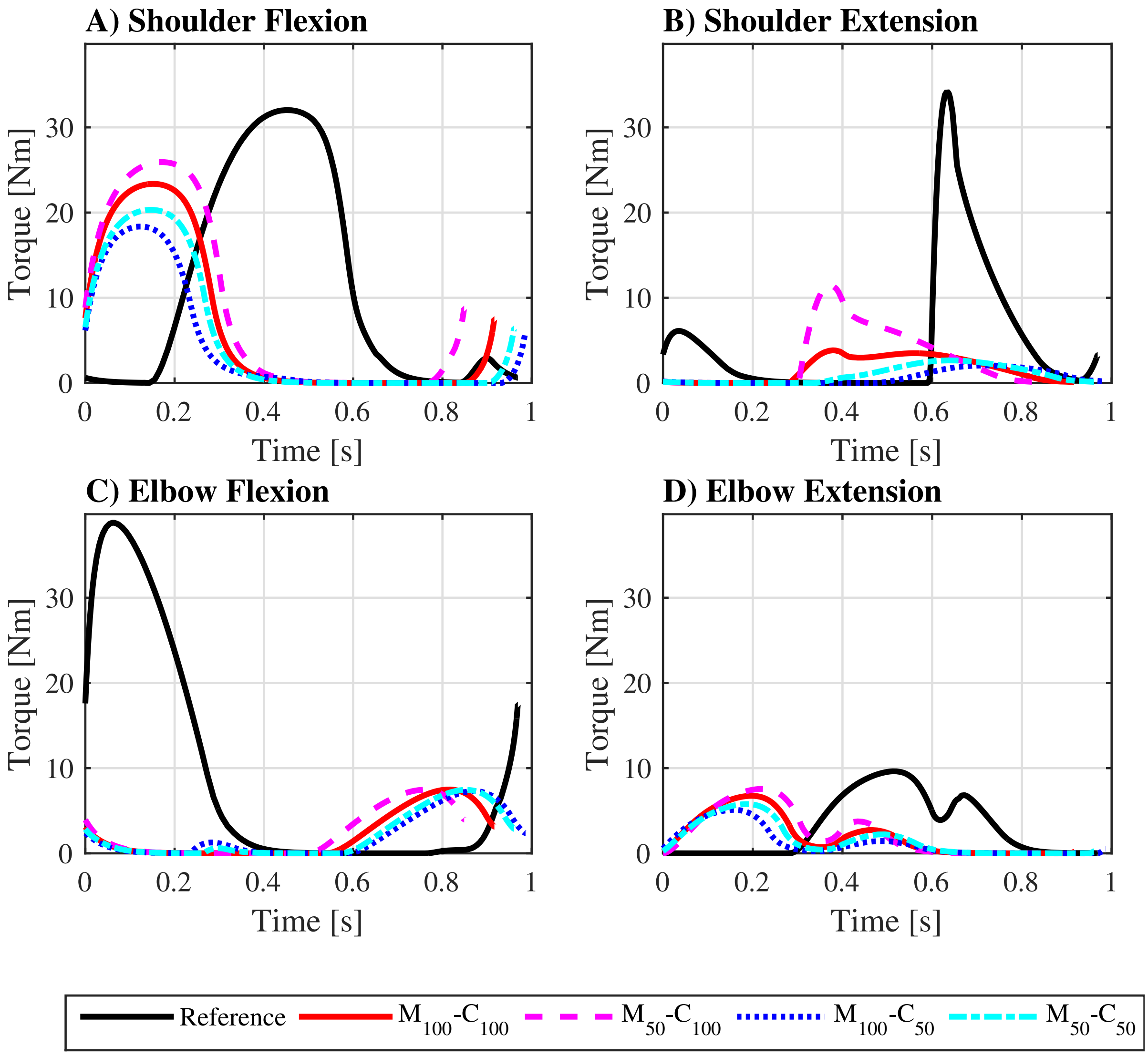

Figure S3. Predicted bilateral shoulder flexion **(A)**, shoulder extension **(B)**, elbow flexion **(C)** and elbow extension **(D)** torque profiles along a complete cycle for *steady state* locomotion on a $3°$ ramp at an average speed of 0.9 m/s for the reference unassisted condition (Reference), and for the assisted locomotion with 100% of reference model parameters M_i and C_i ($M_{100} - C_{100}$), reduction of 50% in M_i ($M_{50} - C_{100}$), reduction of 50% in C_i ($M_{100} - C_{50}$), and reduction of 50% in M_i and C_i ($M_{50} - C_{50}$).

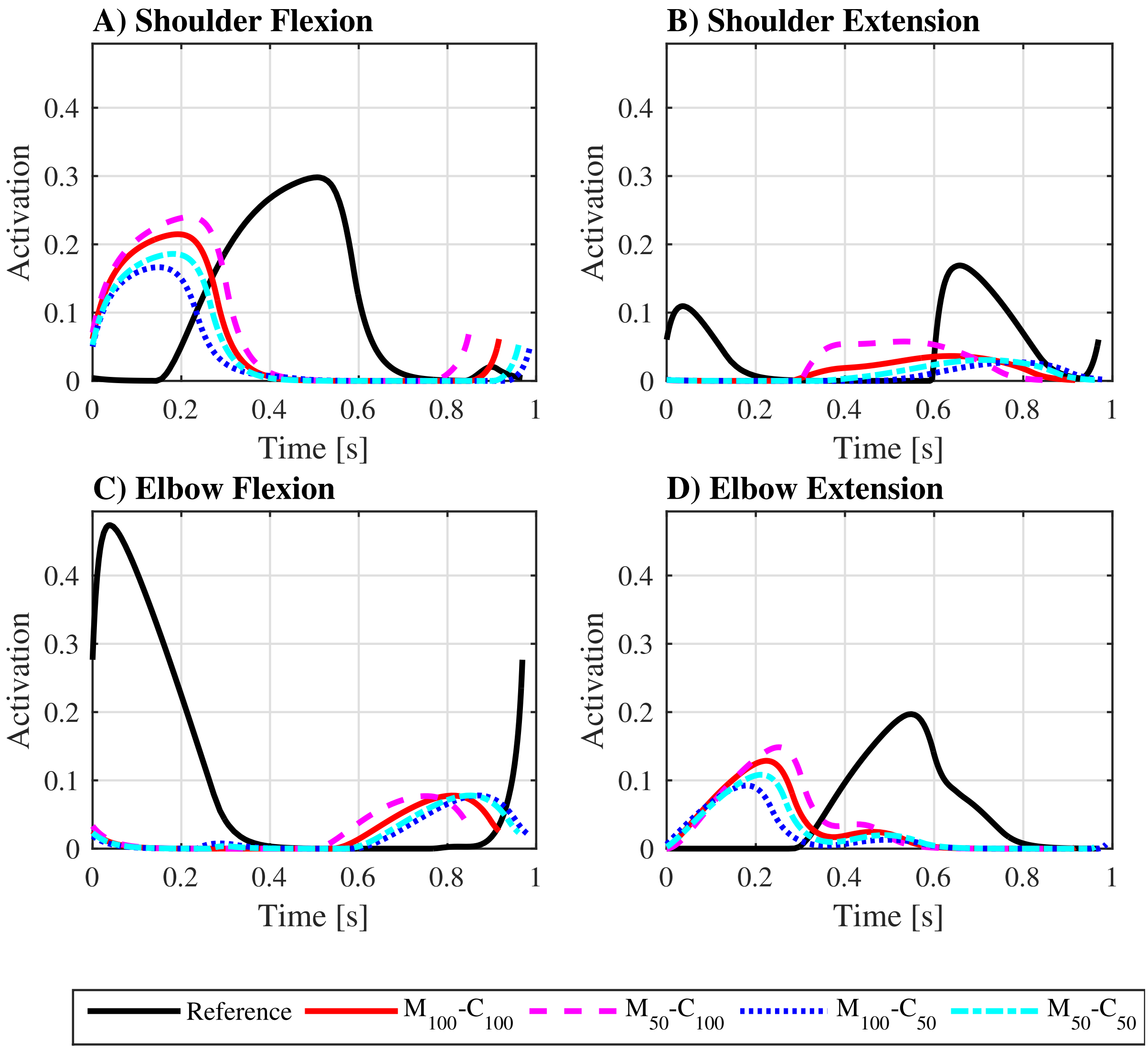

Figure S4. Predicted bilateral shoulder flexion **(A)**, shoulder extension **(B)**, elbow flexion **(C)** and elbow extension **(D)** activation profiles along a complete cycle for *steady state* locomotion on a 3° ramp at an average speed of 0.9 m/s for the reference unassisted condition (Reference), and for the assisted locomotion with 100% of reference model parameters M_i and C_i ($M_{100} - C_{100}$), reduction of 50% in M_i ($M_{50} - C_{100}$), reduction of 50% in C_i ($M_{100} - C_{50}$), and reduction of 50% in M_i and C_i ($M_{50} - C_{50}$).

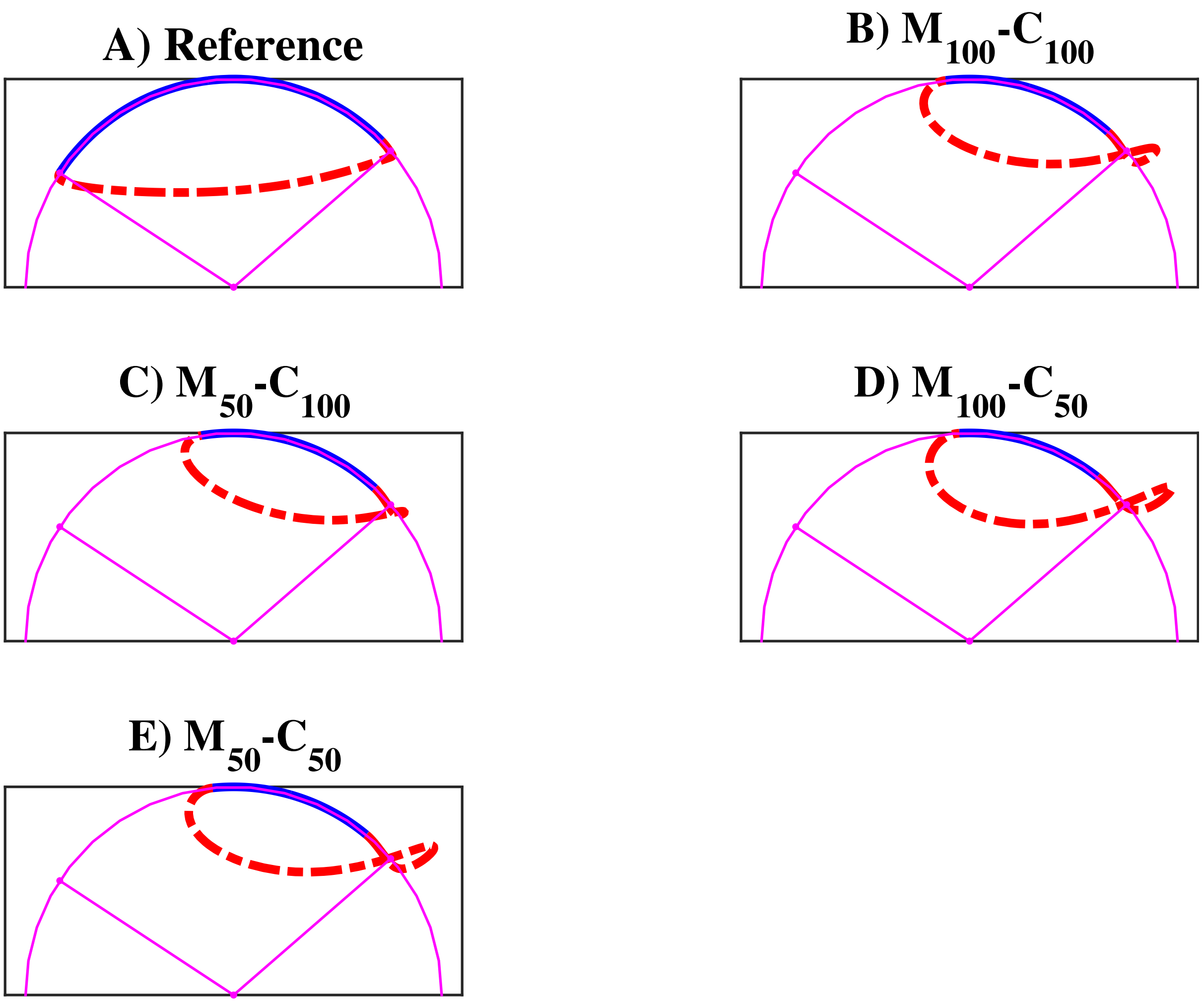

Figure S5. Predicted hand patterns for the reference unassisted condition (Reference) **(A)**, for the assisted locomotion with 100% of reference model parameters M_i and C_i ($M_{100} - C_{100}$) **(B)**, reduction of 50% in M_i ($M_{50} - C_{100}$) **(C)**, reduction of 50% in C_i ($M_{100} - C_{50}$) **(D)**, and reduction of 50% in M_i and C_i ($M_{50} - C_{50}$) **(E)** along a complete cycle for *steady state* locomotion on a $3°$ ramp at an average speed of 0.9 m/s.

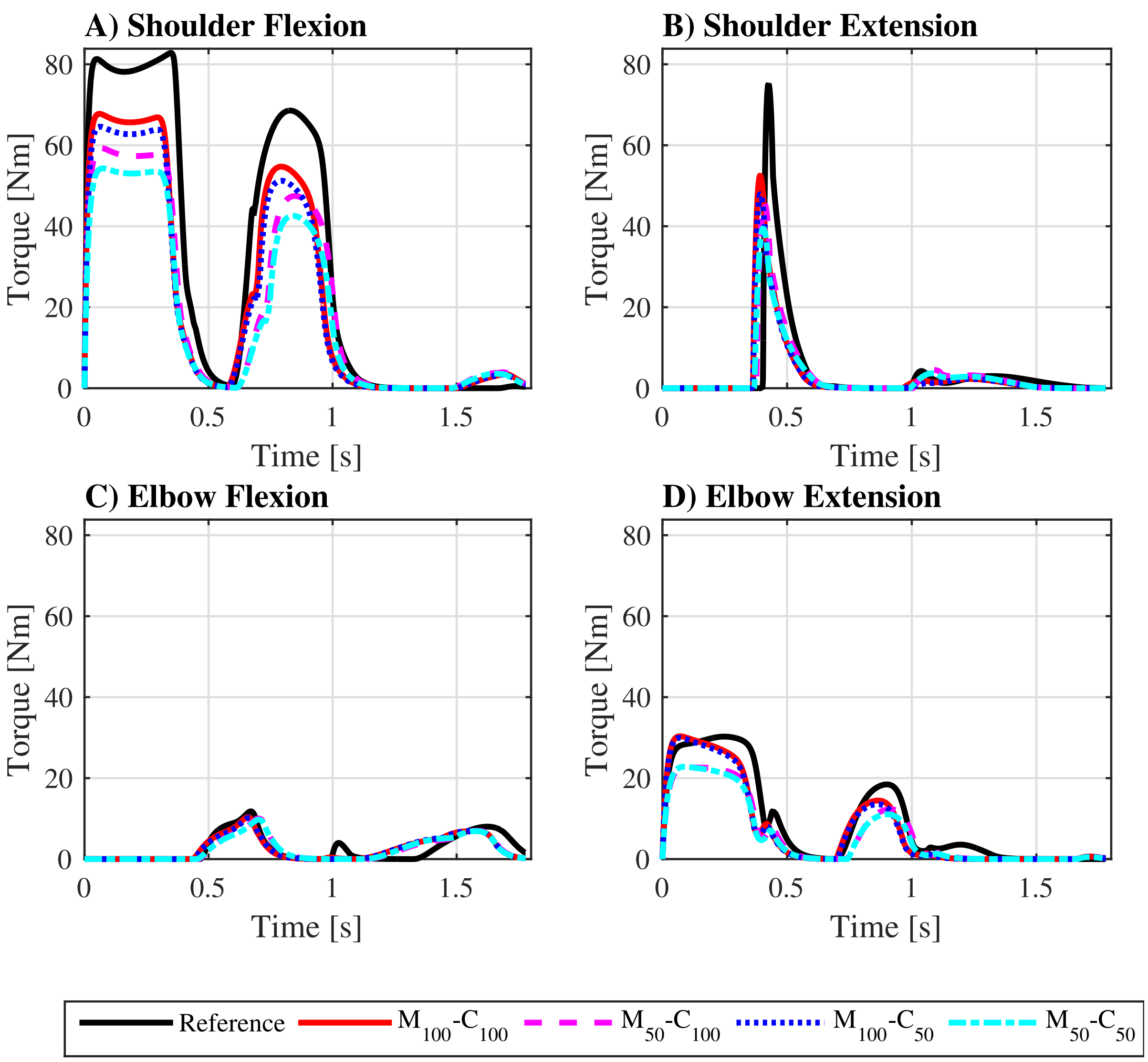

Figure S6. Predicted bilateral shoulder flexion **(A)**, shoulder extension **(B)**, elbow flexion **(C)** and elbow extension **(D)** torque profiles along the sequence of phases propulsion-recovery-propulsion-recovery-propulsion in the *startup* locomotion on a 3° ramp at an average speed of 0.9 m/s for the reference unassisted condition (Reference), and for the assisted locomotion with 100% of reference model parameters M_i and C_i ($M_{100} - C_{100}$), reduction of 50% in M_i ($M_{50} - C_{100}$), reduction of 50% in C_i ($M_{100} - C_{50}$), and reduction of 50% in M_i and C_i ($M_{50} - C_{50}$).

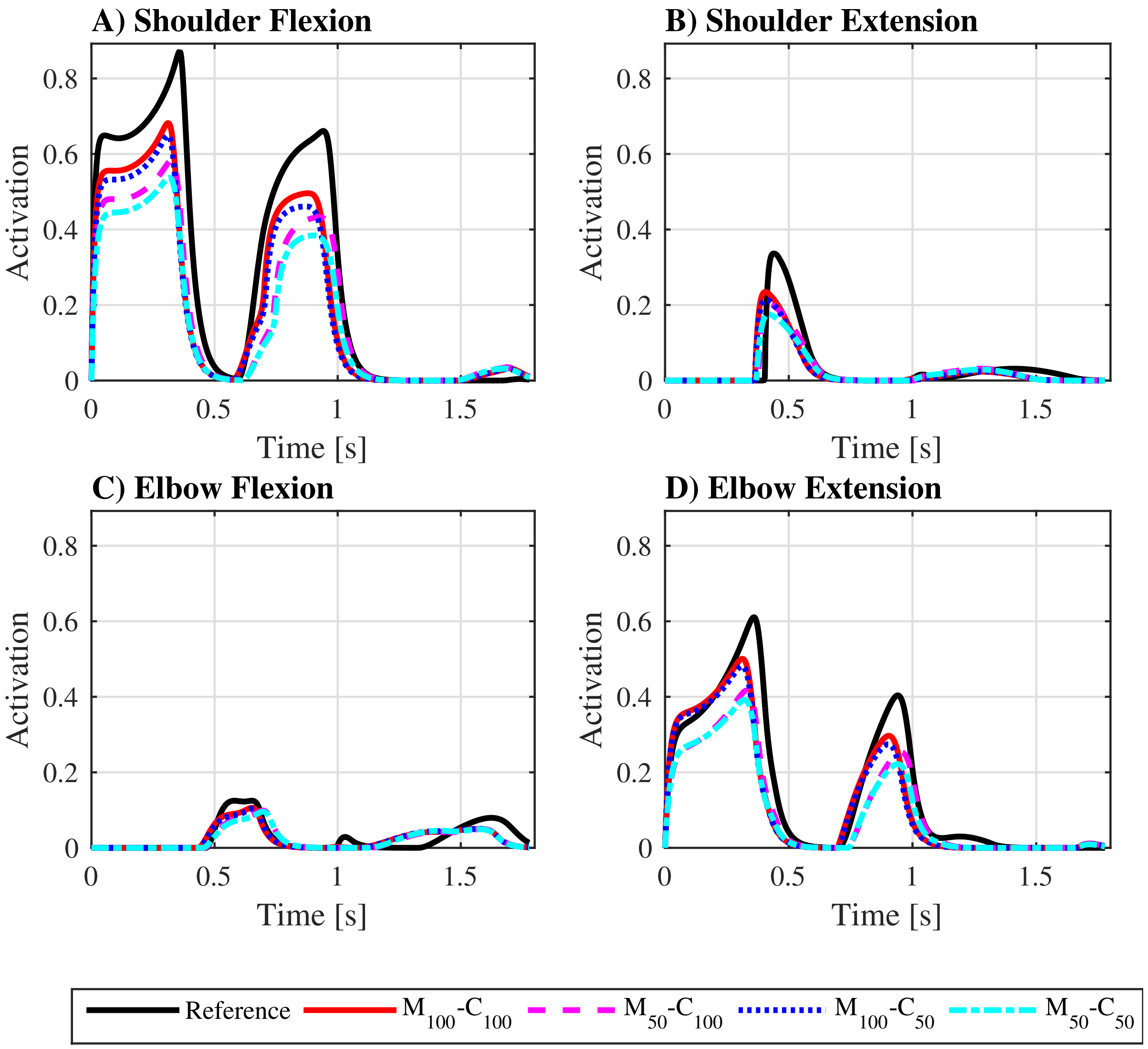

Figure S7. Predicted shoulder flexion **(A)**, shoulder extension **(B)**, elbow flexion **(C)** and elbow extension **(D)** activation profiles along the sequence of phases propulsion-recovery-propulsion-recovery-propulsion in the *startup* locomotion on a 3° ramp at an average speed of 0.9 m/s for the reference unassisted condition (Reference), and for the assisted locomotion with 100% of reference model parameters M_i and C_i ($M_{100} - C_{100}$), reduction of 50% in M_i ($M_{50} - C_{100}$), reduction of 50% in C_i ($M_{100} - C_{50}$), and reduction of 50% in M_i and C_i ($M_{50} - C_{50}$).

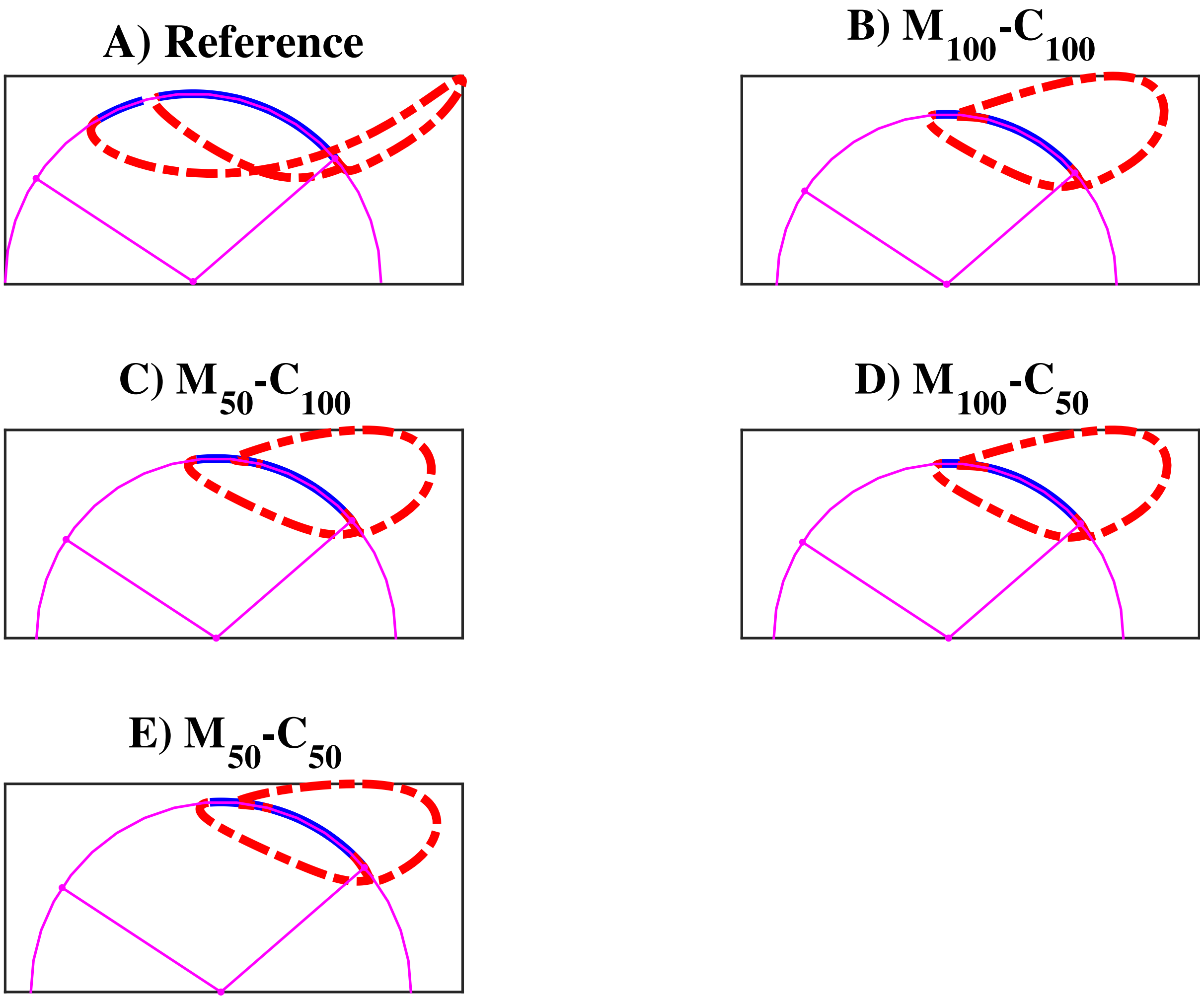

Figure S8. Predicted hand patterns for the reference unassisted condition (Reference) **(A)**, for the assisted locomotion with 100% of reference model parameters M_i and C_i ($M_{100} - C_{100}$) **(B)**, reduction of 50% in M_i ($M_{50} - C_{100}$) **(C)** and reduction of 50% in C_i ($M_{100} - C_{50}$) **(D)**, and reduction of 50% in M_i and C_i ($M_{50} - C_{50}$) **(E)** along the sequence of phases propulsion-recovery-propulsion-recovery-propulsion in the *startup* locomotion on a $3°$ ramp at an average speed of 0.9 m/s.

2 REPOSITORIES

The function relating joint torques with muscle activations, containing force-length, force-velocity relationships and passive joint torques based on Brown (2018), is made available in https://github.com/Vinicius-Ishimoto/Framework-for-Wheelchair-OC-Simulations/blob/main/Main%20Functions/muscle_torque_generators.m.

The results of this study are made available in https://github.com/Vinicius-Ishimoto/Framework-for-Wheelchair-OC-Simulations/tree/main/Results, which is a MATLAB structure with all the data related to the person, wheelchair and the predicted states and controls for all the simulations. Please, follow the README instruction to access the data.
